# Predicting Multiple Types of Associations Between miRNAs and Diseases Based on Graph Regularized Weighted Tensor Decomposition

**DOI:** 10.3389/fbioe.2022.911769

**Published:** 2022-07-04

**Authors:** Dong Ouyang, Rui Miao, Jianjun Wang, Xiaoying Liu, Shengli Xie, Ning Ai, Qi Dang, Yong Liang

**Affiliations:** ^1^ Faculty of Information Technology, Macau University of Science and Technology, Macau, China; ^2^ School of Mathematics and Statistics, Southwest University, Chongqing, China; ^3^ Computer Engineering Technical College, Guangdong Polytechnic of Science and Technology, Zhuhai, China; ^4^ Institute of Intelligent Information Processing, Guangdong University of Technology, Guangzhou, China; ^5^ Peng Cheng Laboratory, Shenzhen, China

**Keywords:** multiple types of miRNA–disease associations, weighted tensor decomposition, graph Laplacian regularization, L2, 1 norm, multi-view biological similarity network

## Abstract

Many studies have indicated miRNAs lead to the occurrence and development of diseases through a variety of underlying mechanisms. Meanwhile, computational models can save time, minimize cost, and discover potential associations on a large scale. However, most existing computational models based on a matrix or tensor decomposition cannot recover positive samples well. Moreover, the high noise of biological similarity networks and how to preserve these similarity relationships in low-dimensional space are also challenges. To this end, we propose a novel computational framework, called WeightTDAIGN, to identify potential multiple types of miRNA–disease associations. WeightTDAIGN can recover positive samples well and improve prediction performance by weighting positive samples. WeightTDAIGN integrates more auxiliary information related to miRNAs and diseases into the tensor decomposition framework, focuses on learning low-rank tensor space, and constrains projection matrices by using the *L*_2,1_ norm to reduce the impact of redundant information on the model. In addition, WeightTDAIGN can preserve the local structure information in the biological similarity network by introducing graph Laplacian regularization. Our experimental results show that the sparser datasets, the more satisfactory performance of WeightTDAIGN can be obtained. Also, the results of case studies further illustrate that WeightTDAIGN can accurately predict the associations of miRNA–disease-type.

## 1 Introduction

MicroRNAs (miRNAs) are small non-coding RNA molecules with a length of about 22–24 nucleotides, which can regulate gene expression and protein synthesis at the post-transcriptional level (Ambros, 2004; Bartel, 2004; Bushati and Cohen, 2007). To be more specific, miRNAs can affect protein synthesis by promoting or inhibiting gene expression, thereby causing the occurrence and development of diseases. In addition, a great number of studies have shown that the mutation or abnormal expression of miRNAs often leads to the occurrence of many complex human diseases. For example, hsa-mir-195 and hsa-mir-497 have been shown to play a key inhibitory role in breast cancer malignancies, which can even become potential diagnostic targets (Li et al., 2011). It is exciting that mir-375 can regulate the secretion of insulin (Poy et al., 2009). Thus, identifying the potential miRNA–disease associations will help us understand the molecular mechanisms of miRNA-related diseases and provide a new way to treat diseases. Moreover, the discovery of disease-related miRNAs will contribute to the study of disease pathological mechanisms from a deeper perspective and the identification of potential disease biomarkers (Chen et al., 2019a). At present, traditional experimental methods used to identify potential miRNA–disease associations mainly include reverse transcription-polymerase chain reaction (Freeman et al., 1999), Northern blotting (Várallyay et al., 2008), and microarray profiling (Baskerville and Bartel, 2005). However, such traditional experimental methods, requiring a lot of time and money investment, are often inefficient and prone to failure easily. Therefore, there is an urgent need for more and more computational methods that can provide supporting pieces of evidence and more efficient predictions to accelerate the diagnosis and treatment of human diseases.

According to previous studies (Zeng et al., 2016), existing calculation methods can be divided into two categories: similarity measure-based methods and machine learning-based methods. Among them, similarity-based methods to predict the potential associations of miRNA–disease are based on the assumption that miRNAs with similar functions are more likely to be related to similar diseases. Chen et al. (2018) discovered potential miRNA–disease associations by integrating the predicted association probability obtained from matrix decomposition and similarity information related to miRNAs and diseases into heterogeneous networks. Cui et al. (2019) designed a method based on a bipartite local model with nearest profile-based association inferring, which can predict the association through its nearest neighbor even without any association. Yin et al. (2020) proposed a method based on network consistency projection and label propagation, which obtains network projection scores by using network consistency projection and label propagation is utilized for association prediction. Li et al. (2021a) used a similarity network fusion algorithm to integrate the similarity of multiple miRNAs and diseases, and graph Laplacian regularization was constrained to matrix factorization to predict miRNA–disease associations. Zhou et al. (2021) utilized multiple kernel learning to construct similarity networks between miRNA and disease, and a regression model was used to learn feature representation based on these networks. Then, these feature representations are input into a deep autoencoder to predict miRNA–disease associations. Machine learning-based methods have been proposed to better extract features, which can more accurately predict the associations between miRNAs and diseases. Chen et al. (2019b) integrated ensemble learning and dimensionality reduction based on principal component analysis for inferring potential miRNA–disease associations. Li et al. (2021b) used a graph convolutional autoencoder to calculate association scores based on the two sub-networks of miRNAs and diseases in a heterogeneous network and adopted an average ensemble method to obtain the final prediction score. Li et al. (2021c) proposed a novel graph autoencoder method named GAEMDA to predict the potential miRNA–disease associations in an end-to-end manner. Tang et al. (2021) utilized a graph convolutional network and attention mechanism to extract and enhance the latent representations of miRNA and disease in multiple views for reconstructing the miRNA–disease association matrix. Yan et al. (2022) developed a new end-to-end deep learning method named PDMDA, which utilizes a fully connected network and graph neural network to extract the feature representations of miRNAs and diseases for deep-level miRNA–disease association prediction.

Based on the previous research works, these methods only focus on miRNA–disease binary association prediction, without considering the specific type of miRNA. However, more and more experimental evidence shows that the mechanism of miRNAs causing diseases is very complex, rather than a simple binary association prediction (Fabbri et al., 2007; Vogt et al., 2011; He et al., 2017). On the one hand, miRNAs are related to diseases, but diseases are only caused by the specific type of miRNA. For example, the mir-29 family (29a, b, c) reverses the abnormal methylation of lung cancer by targeting DNA methyltransferases 3A and 3B (Fabbri et al., 2007). On the other hand, the mechanism of the same miRNA causing the same disease is distinguished in different types. For instance, the occurrence of CpG methylation leads to epigenetic inactivation of mir-34a in breast cancer, meanwhile, circGFRA1 may be a potential target in triple-negative breast cancer by regulating mir-34a (Vogt et al., 2011; He et al., 2017). Therefore, while predicting the potential associations between miRNAs and diseases, we also need to determine which types of miRNAs are related to the diseases.

In the past few years, some researchers have focused on the problem of identifying multiple types of miRNA–disease associations. Chen et al. (2015) are the first to study the problem of multiple types of miRNA–disease associations, which provides a new idea for researchers to understand the pathogenesis of diseases in more detail at the molecular level. In their study, they developed a restricted Boltzmann machine model for multiple types of miRNA–disease association prediction (RBMMMDA). Zhang et al. (2018) proposed a semi-supervised model called a network-based label propagation algorithm to predict multiple types of miRNA–disease association (NLPMMDA), and multiple-view of miRNA and disease information was integrated into a heterogeneous network. However, these models either did not consider the auxiliary information related to miRNAs and diseases or ignored the inherent connection of the multiple-type association matrices. Fortunately, tensor, as a multi-dimensional array, can well represent multiple-type miRNA–disease associations as a triplet. Biological similarity information as decomposition constraints can also be incorporated into the framework of tensor decomposition to explore some unobserved triples by decomposing a tensor. Huang et al. (2021) integrated miRNA functional similarity and disease semantic similarity as auxiliary information into tensor decomposition and proposed a tensor decomposition with relational constraint (TDRC) model. However, TDRC does not recover positive samples well, and its prediction performance has not been effectively improved. Next, although TDRC takes miRNA-miRNA functional similarity and disease-disease semantic similarity information as decomposition constraints, the computing framework cannot easily expand the information related to miRNAs and disease to effectively solve the problem of tensor sparseness and further improve the performance of the model. Moreover, TDRC cannot effectively avoid learning irrelevant information in the training stage. Finally, TDRC does not well preserve the similarity relationships of internal nodes between diseases and between miRNAs.

To address the aforementioned problems, in this article, we propose a computational framework named **Weight**ed **T**ensor **D**ecomposition with **A**uxiliary **I**nformation, **G**raph Laplacian regularization, and *L*_2,1_ **N**orm (WeightTDAIGN), which integrates weight, graph Laplacian regularization, *L*_2,1_ norm, and more auxiliary information into tensor decomposition to better predict multiple types of miRNA–disease associations. First, WeightTDAIGN can recover positive samples well and improve prediction performance by weighting positive samples. Second, WeightTDAIGN can incorporate more miRNA-related and disease-related auxiliary information through changing the interactive update strategy of factor matrices and biological similarity matrices. Furthermore, features that contain more information can be learned by constraining projection matrices using the *L*_2,1_ norm, which can effectively avoid learning the noise information in the biological similarity network. Next, to make better use of the known biological similarity networks, we introduce graph Laplacian regularization to capture the data geometric structure between biological similarity networks. Finally, we optimize the framework using an alternate iteration strategy and adopt the alternating direction method of multipliers (ADMM) algorithm to infer multiple types of miRNA–disease associations. The WeightTDAIGN model we proposed is compared with six benchmark models on four datasets with different sparsities. The experimental results show that the WeightTDAIGN model is superior to these models including the latest model TDRC, especially when datasets are sparse. Additionally, the results of case studies demonstrate that WeightTDAIGN can accurately predict the associations of miRNA–disease-type and discover the potential associations of unconfirmed miRNA–disease that are of biological significance, which further validates the effectiveness of the proposed model.

## 2 Materials

### 2.1 Human miRNA–Disease-Type Association Datasets

More miRNA-related disease databases or tools are emerging, which provide convenience for identifying the potential associations between miRNAs and diseases from the perspective of computational methods (Chen et al., 2019a). In this article, we used the HMDD v3.2 and HMDD v2.0 versions of the Human miRNA Disease Database (HMDD) as benchmark datasets for constructing tensors (Wang et al., 2010). HMDD v3.2 and HMDD v2.0 can be downloaded from https://www.cuilab.cn/hmdd. Meanwhile, disease descriptors can be provided from Medical Subject Headings (MeSH). Also, miRNA sequence can be obtained from miRBase (Kozomara and Griffiths-Jones, 2014). HMDD v2.0 is classified into four types based on the evidence from circulation, epigenetics, genetics, and target. Moreover, the recently released HMDD v3.2 provides six generalized types of associations (circulation, epigenetics, genetics, target, tissue, and others). However, the category of miRNA is not clear for the “other” category, so we did not download the data of that category. In addition, we mapped the human miRNA–disease-type associations with experimentally verified into 0 and 1. In detail, if a disease is associated with a miRNA of a certain type, then the value is set as 1, otherwise 0. In order to explore the generalization ability of WeightTDAIGN under different sparsity data, we divided HMDD v3.2 and HMDD v2.0 into four datasets according to the proportion of data sparsity.

Based on previous research (Chen et al., 2016; Pasquier and Gardès, 2016; Liang et al., 2019), we expanded some auxiliary information, such as miRNA sequence similarity and Gaussian interaction profile kernel similarity for miRNAs and diseases, to further improve the prediction performance. For miRNAs, we only retained these miRNAs that can get the sequence from miRBase. For diseases, we deleted the diseases which are not found and whose category is not C for the tree structure in MeSH. The detailed descriptions of the four datasets are shown in Table 1.

**TABLE 1:** Statistics of all datasets used in this study.

Dataset	#miRNA	#Disease	#Type	#Association	#Density (%)
MDA v2.0-2	211	59	4	1,410	2.83
MDA v2.0-3	69	25	4	586	8.49
MDA v2.0-4	40	20	4	347	10.84
MDA v3.2-5	125	65	5	4,785	11.78

#Disease, disease number; #miRNA, miRNA number; #asssociation, association number; #type, type number; #density, sparsity rate.

After aforementioned preprocessing and removing duplications, we finally obtained the following datasets:

• MDA v2.0–2 is obtained from HMDD v2.0 released in 2013. We removed these miRNAs or diseases that involve less than two associations in total across all types.
• MDA v2.0–3 also deleted miRNAs or diseases that include less than three associations in total across all types for HMDD v2.0.
• MDA v2.0–4 is also obtained from HMDD v2.0. We only got miRNAs and diseases that exist in total across all types.
• MDA v3.2–5 is released from HMDD v3.2, which contains five-type (circulation, epigenetics, genetics, target, and tissue) association matrices and only includes miRNAs and diseases that exist in total across all types.

### 2.2 Tensor Construction

Given a set of miRNA–disease associations $\mathrm{MD}=\left\{\left(m_{1},d_{1}\right),\left(m_{1},d_{2}\right),\ldots ,\left(m_{\left|m\right|},d_{\left|n\right|}\right)\right\}$ and a set of association types $T=\left\{t_{1},t_{2},\ldots ,t_{\left|t\right|}\right\}$ , we can construct a binary third-order tensor $X\in {\left\{0,1\right\}}^{\left|m|\times |n|\times |t\right|}$ in the form of fiber, where |*m*|, |*n*|, and |*t*| represent the size of the set of miRNAs, diseases, and types, respectively. Obviously, one of the tensor entries *x*_*ijt*_ is set to 1 if a type of miRNA is related to the disease. Otherwise, the entries are set to 0. When the dimension representing the type of a tensor is fixed, each slice of a tensor refers to a type of miRNA–disease association. Our goal is to infer potential multiple types of miRNA–disease associations through tensor completion. The third-order tensor is sparse with many unknown entries, which may influence the prediction performance of the model. To overcome this problem, multi-view of miRNA and disease similarity as auxiliary information is considered.

### 2.3 Disease Semantic Similarity

Based on the research of Wang et al. (2010), disease semantic similarity can be computed by MeSH descriptors, which can be obtained from https://www.ncbi.nlm.nih.gov/. Herein, the relationships of different diseases can be represented by directed acyclic graphs (DAGs). We adopted *DAG* (*d*_*i*_) = (*T* (*d*_*i*_), *E* (*d*_*i*_)) to describe the relationships of all diseases, in which *T* (*d*_*i*_) and *E* (*d*_*i*_) denote the node set and edge set, respectively. For a node *d*_*i*_, it represents a disease. Also, a set of edges *E* (*d*_*i*_) represents the relationships between different diseases. Then, we can calculate the semantic contribution of disease *d*_*t*_ to *d*_*i*_ as follows:

$$\begin{array}{ll} D1\left(d_{i},d_{t}\right) & =\left\{\begin{array}{cc} 1,\; & \;\mathrm{if}d_{t}=d_{i}\\ \max\left\{\Delta *D1\left(d_{i},d_{t}^{\prime }\right)|d_{t}^{\prime }\in children\;of\;d_{t}\right\},\; & \;\mathrm{if}d_{t}\neq d_{i}, \end{array}\right. \end{array}$$ (1)

where Δ represents the semantic contribution decay factor, and we set Δ = 0.5 according to previous study (Wang et al., 2010). The semantic contribution value of diseases *d*_*i*_ and *d*_*t*_ can be described as the distance between them. Thus, the semantic value of disease *d*_*i*_ can be defined as follows:

$$SV1\left(d_{i}\right)=\underset{d_{t}\in T\left(d_{i}\right)}{\sum }D1\left(d_{i},d_{t}\right).$$ (2)

Based on Eqs 1, 2, we can obtain the disease semantic similarity *DSS*1(*d*_*i*_, *d*_*j*_) between diseases *d*_*i*_ and *d*_*j*_ as follows:

$$DSS1\left(d_{i},d_{j}\right)=\frac{\sum_{d_{k}\in T\left(d_{i}\right)\cap T(d_{j})}\left(D1\left(d_{i},d_{k}\right)+D1\left(d_{j},d_{k}\right)\right)}{SV1\left(d_{i}\right)+SV1\left(d_{j}\right)}.$$ (3)

Moreover, it is worth noting that diseases may be more common in more DAGs and more specific in fewer DAGs. Therefore, the disease semantic contribution value of the same layer should be different in DAGs. Then, based on a previous study (Pasquier and Gardès, 2016), we applied another method to calculate the semantic contribution of disease *d*_*t*_ to *d*_*i*_ as shown below:

$$D2\left(d_{i},d_{t}\right)=-\log\left(\frac{the\;number\;of\;DAGs\;including\;d_{t}}{the\;number\;of\;disease}\right).$$ (4)

Calculation ideas based on Eqs 3, 4, we can obtain the semantic value *SV*2(*d*_*i*_) of disease *d*_*i*_ and the disease semantic similarity *DSS*2(*d*_*i*_, *d*_*j*_) between disease *d*_*i*_ and *d*_*j*_ as follows:

$$SV2\left(d_{i}\right)=\underset{d_{t}\in T\left(d_{i}\right)}{\sum }D2\left(d_{i},d_{t}\right),$$ (5)

$$DSS2\left(d_{i},d_{j}\right)=\frac{\sum_{d_{k}\in T\left(d_{i}\right)\cap T(d_{j})}\left(D2\left(d_{i},d_{k}\right)+D2\left(d_{j},d_{k}\right)\right)}{SV2\left(d_{i}\right)+SV2\left(d_{j}\right)}.$$ (6)

In order to acquire a more reasonable semantic similarity of diseases, we can calculate the final disease semantic similarity *DSS*(*d*_*i*_, *d*_*j*_) between disease *d*_*i*_ and *d*_*j*_ according to the following equation:

$$DSS\left(d_{i},d_{j}\right)=\frac{DSS1\left(d_{i},d_{j}\right)+DSS2\left(d_{i},d_{j}\right)}{2}.$$ (7)

### 2.4 MiRNA Functional Similarity

Based on the assumption that miRNAs with similar functions are more likely to induce similar diseases, the miRNA functional similarity score can be calculated by Wang et al. (2010). According to miRNA functional similarity information, we can build an *M* × *M* matrix *MFS*. *M* refers to the number of miRNAs. *MFS*(*m*_*i*_, *m*_*j*_) denotes each element in the matrix *MFS*, which also represents the miRNA functional similarity score between miRNAs *m*_*i*_ and *m*_*j*_.

$$MFS\left(m_{i},m_{j}\right)=\frac{\underset{d\in D\left(m_{i}\right)}{\sum }\;DSS\left(d,d_{j}^{*}\right)\;+\;\underset{d\in D\left(m_{j}\right)}{\sum }\;DSS\left(d,d_{i}^{*}\right)}{\left|D\left(m_{i}\right)|+|D\left(m_{j}\right)\right|},$$ (8)

where *D* (*m*_*i*_) denotes the set of diseases that are associated with *m*_*i*_ in at least one association type and |*D* (*m*_*i*_)| is the number of elements in the set *D* (*m*_*i*_) and $d_{i}^{*}=\underset{d_{i}\in D\left(m_{i}\right)}{argmax}DSS\left(d,d_{i}\right)$ .

### 2.5 MiRNA Sequence Similarity

According to the description of Liang et al. (2019), we utilized the “pairwiseAlignment” function in the R package “Biostrings” to calculate the sequence similarity scores of miRNAs. Finally, we achieved the miRNA sequence similarity matrix *MSS* by min-max normalization as follows:

$$MSS\left(m_{i},m_{j}\right)=\frac{Score\left(m_{i},m_{j}\right)-Score_{\min}}{Score_{\max}-Score_{\min}},$$ (9)

where *Score* *_min_* and *Score* *_max_* represent the maximum and minimum values in the similarity score matrix *Score*, respectively. The *Score* can be calculated by using the “pairwiseAlignment” function.

### 2.6 Gaussian Interaction Profile Kernel Similarity for miRNAs and Diseases

Based on previous research (Van Laarhoven et al., 2011), we assumed that miRNAs with similar functions have similar interaction and non-interaction patterns with diseases and leveraged the Gaussian kernel to extract nonlinear information related to miRNAs and diseases from known miRNA–disease associations as the Gaussian interaction profile kernel similarity. Correspondingly, a binary vector *IP*(*m*_*i*_) refers to whether miRNA *m*_*i*_ is associated with each disease in the known miRNA–disease association datasets. Then, the Gaussian interaction profile kernel similarity between miRNA *m*_*i*_ and *m*_*j*_ can be calculated as follows:

$$MGPS\left(m_{i},m_{j}\right)=\exp\left(-\gamma_{m}\|IP\left(m_{i}\right)-IP\left(m_{j}\right){\|}^{2}\right),$$ (10)

where the parameter *γ*_*m*_ controls the bandwidth of kernel. It can be expressed as a normalization of the average number of associations between miRNAs and diseases. The formulation for the calculation of *γ*_*m*_ is shown below:

$$\gamma_{m}=\frac{\gamma_{m}^{\prime }}{\frac{1}{nm}\sum_{i=1}^{nm}\|IP\left(m_{i}\right){\|}^{2}},$$ (11)

where *nm* represents the number of all miRNAs. Herein, we set $\gamma_{m}^{\prime }=1$ according to the previous work (Chen et al., 2016). Similarly, the Gaussian interaction profile kernel similarity between diseases *d*_*i*_ and *d*_*j*_ can be calculated as follows:

$$DGPS\left(d_{i},d_{j}\right)=\exp\left(-\gamma_{d}\|IP\left(d_{i}\right)-IP(d_{j}){\|}^{2}\right),$$ (12)

$$\gamma_{d}=\frac{\gamma_{d}^{\prime }}{\frac{1}{nd}\sum_{i=1}^{nd}\|IP\left(d_{i}\right){\|}^{2}},$$ (13)

where a binary vector *IP*(*d*_*i*_) refers to whether disease *d*_*i*_ is associated with each miRNA in the known miRNA–disease associations datasets. *nd* denotes the number of all diseases, and $\gamma_{d}^{\prime }$ is also set to 1.

## 3 Methods

### 3.1 CP Decomposition

CANDECOMP/PARAFAC (CP) decomposition is one of the most common tensor decomposition forms (Kolda and Bader, 2009). Given the miRNA-disease-type tensor $X\in R^{\left|m|\times |n|\times |t\right|}$ , the CP decomposition model can be represented as follows:

$$X\approx \overset{S}{\underset{s=1}{\sum }}m_{s}◦d_{s}◦t_{s}\equiv \left[\left[M,D,T\right]\right]$$ (14)

where the symbol ◦ represents the vector outer product, *S* is a positive integer and $m_{s}\in R^{|m|\times 1}$ , $d_{s}\in R^{|n|\times 1}$ and $t_{s}\in R^{|t|\times 1}$ . *M* = [***m***_1_ ***m***_2_ ⋯ ***m***_*S*_], *D* = [***d***_1_ ***d***_2_ ⋯ ***d***_*S*_], and *T* = [***t***_1_ ***t***_2_ ⋯ ***t***_*S*_] are the factor matrices with respect to different dimensions.

Then, the optimization problem of CP decomposition can be easily considered as follows:

$$\underset{M,D,T}{\min}\|X-\left[\left[M,D,T\right]\right]{\|}_{F}^{2},$$ (15)

where ‖⋅‖_*F*_ is the tensor Frobenius norm. In our work, $M\in R^{|m|\times r}$ , $D\in R^{|n|\times r}$, and $T\in R^{|t|\times r}$ represent the miRNA, disease, and type mode, respectively. Herein, *r* refers to the rank of the approximated tensor [[*M*, *D*, *T*]]. Furthermore, the rank of the tensor is usually set to a smaller constant to obtain a low-rank approximation. The optimization problem Eq. 15 can be solved by the alternating least squares (ALS) method.

### 3.2 Weight Tensor

Based on the research of Huang et al. (2021), the prediction of associated types in the problem of binary association prediction of miRNA–diseases is the focus of our research. However, we observe that the existing methods based on tensor decomposition cannot effectively recover positive samples, which makes it impossible to predict multiple types of miRNA–disease associations more accurately. Therefore, we propose a strategy of weighting positive samples to make them better recovered. Suppose the original tensor is X , the reconstructed tensor is $\tilde{X}$ . Given the weight tensor W , we can obtain the objective function as follows:

$$\underset{\tilde{X}}{\min}\|\sqrt{W}⊛\left(X-\tilde{X}\right){\|}_{F}^{2}+f\left(W\right),$$ (16)

where W⊛Z denotes the element-wise product of tensor W and Z . f(W) determines that positive samples are weighted at the element level.

Obviously, the loss value is highly correlated with the result of positive sample reconstruction. To determine which positive samples are weighted by the change of the loss value, we set f(W) as follows:

$$f\left(W\right)=-\overset{\left|m\right|}{\underset{i=1}{\sum }}\overset{\left|n\right|}{\underset{j=1}{\sum }}\overset{\left|t\right|}{\underset{t=1}{\sum }}{\left(1-k\right)}^{2}W_{\mathrm{ijt}},$$ (17)

where *k* is the artificially set threshold, which controls the range of weights given to positive samples. The range of *k* is between 0 and 1 in this article.

We rewrite Eq. 16 element-wise and find that $W_{{\left(n\right)}_{ij}}$ can be easily solved by the following formulation:

$$W_{{\left(n\right)}_{ij}}=\left\{\begin{array}{cc} 1,\; & \;\mathrm{if}loss\leq {\left(1-k\right)}^{2}\\ w,\; & \;\mathrm{otherwise}. \end{array}\right.$$ (18)

Herein, *w* refers to a weight value and *w* ≥ 1, $loss={\left(X_{{\left(n\right)}_{ij}}-{\tilde{X}}_{{\left(n\right)}_{ij}}\right)}^{2}$ . $X_{\left(n\right)}$ and $W_{\left(n\right)}$ are the mode-*n* matricization of tensor X and W , respectively.

### 3.3 WeightTDAIGN

#### 3.3.1 Model Auxiliary Information

**FIGURE 1:**
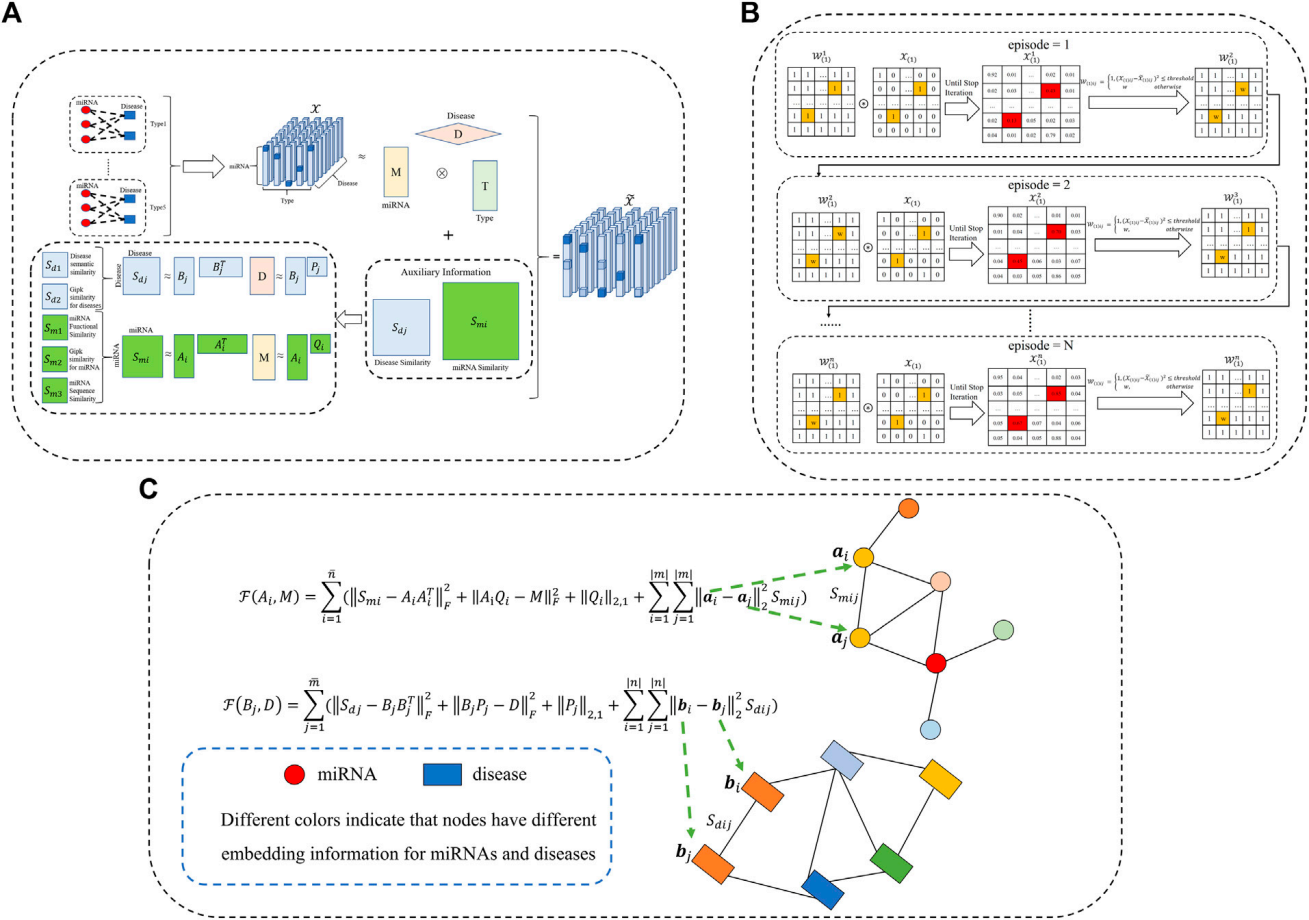
The workflow of our proposed WeightTDAIGN model for predicting potential multiple types of miRNA–disease associations. **(A)** Multi-view miRNA and disease similarity networks are incorporated into tensor decomposition. It is worth noting that Gipk represents the Gaussian interaction profile kernel. **(B)** We take slice $W_{\left(1\right)}$ as an example to show how to assign weight to positive samples. **(C)** If the similarity *S*_*mij*_ between miRNAs (or diseases) is high, the embedding information of nodes **a**_*i*_ and **a**_*j*_ will be very similar (that is, the nodes have the same color) for miRNAs (or diseases) in the low-dimensional embedding space.

To leverage the existing prior knowledge of miRNA-miRNA and disease-disease similarity networks, we utilize matrix factorization to learn important latent information about the similarity matrix. Inspired by previous research (Chen and Li, 2017, 2018), the multi-view information for miRNAs and diseases should share the same latent structures. Consequently, we change the interactive update strategy of factor matrices and similarity matrices, which can fuse more biological similarity information into the calculation framework. The whole workflow of the proposed WeightTDAIGN model is presented in Figure 1. Given multiple views of miRNAs (or diseases) similarity matrices $S=\left\{S_{m1},S_{m2},\ldots ,S_{m\bar{n}}\right\}$ , the objective function can be defined as follows:

$$F\left(A_{i},A^{*}\right)=\overset{\bar{n}}{\underset{i=1}{\sum }}\left(\|S_{mi}-A_{i}A_{i}^{T}{\|}_{F}^{2}+\|A_{i}Q_{i}-A^{*}{\|}_{F}^{2}\right),$$ (19)

where $\bar{n}$ is the number of miRNA similarity views; *S*_*mi*_ represents the miRNA similarity matrix for *i*th view; $A_{i}\in R^{|m|\times r^{\prime }}$ is the miRNA latent factor for *i*th view, and *r*′ is the low-dimensional embedding representation (*r*′ ≪|*m*|); $A^{*}\in R^{|m|\times r}$ is the common latent structure shared by all views related to miRNAs; and $Q_{i}\in R^{r^{\prime }\times r}$ is the projection matrix.

#### 3.3.2 Model Constraint Information

Recent studies have shown that data distributed in high-dimensional space often contain important local information, and preserving this local structure information of data can improve the performance of the model when high-dimensional data is embedded into low-dimensional subspace (Meng et al., 2018; Shang et al., 2020). Given a miRNA (or disease) similarity matrix *S*_*m*_, *A* is defined as the low-dimensional embedding matrix of the high-dimensional data *S*_*m*_. To preserve the local structure information of high-dimensional data, the objective function to be solved is as follows:

$$\arg\min_{A}\overset{\left|m\right|}{\underset{i=1}{\sum }}\overset{\left|m\right|}{\underset{j=1}{\sum }}\|a_{i}-a_{j}{\|}_{2}^{2}S_{\mathrm{mij}}=\mathrm{Tr}\left(A^{T}LA\right),$$ (20)

where ***a***_*i*_ refers to the *i*th row vector of the low-dimensional embedding matrix *A*. It can be seen from Eq. 20 that if the similarity *S*_*mij*_ between miRNAs *i* and *j* is high, the distance between the embedding information ***a***_*i*_ and ***a***_*j*_ should be very close. *L*_*m*_ = *D*_*m*_ − *S*_*m*_ and *D*_*m*_ is the degree matrix and $D_{\mathrm{mii}}=\sum_{j=1}^{\left|m\right|}S_{\mathrm{mij}}$ .

When the information of the multiple views of miRNA (or disease) similarity network is projected to a common latent matrix, the irrelevant feature information is usually included. To discard irrelevant features and make the model select features with more information (Nie et al., 2010), the *L*_2,1_ norm is constrained on the projection matrix *Q*.

$$\|Q{\|}_{2,1}=\overset{r^{\prime }}{\underset{i=1}{\sum }}\sqrt{\overset{r}{\underset{j=1}{\sum }}q_{ij}^{2}}=\overset{r^{\prime }}{\underset{i=1}{\sum }}\|q^{i}{\|}_{2},$$ (21)

where **q** ^*i*^ denotes the *i*th row of the projection matrix *Q*.

#### 3.3.3 WeightTDAIGN: Optimization Formulation

To consider the biological similarity network in the tensor decomposition and preserve the local structural information of this network, we combine Eqs 19 and 20 with the weighted tensor decomposition Eq. 16. Moreover, to alleviate the introduction of noise information and prevent overfitting, we use the *L*_2,1_ norm to constrain the projection matrices and add the *L*_2_ regularization term for factor matrices. Finally, the objective function can be obtained as follows:

$$\begin{array}{ll} & \underset{M,D,T,W,A,B,Q,P}{\min}\|\sqrt{W}⊛\left(X-\left[\left[M,D,T\right]\right]\right){\|}_{F}^{2}\\ & \;+\sum_{i=1}^{\bar{n}}\alpha_{i}\left(\|S_{mi}-A_{i}A_{i}^{T}{\|}_{F}^{2}+\|A_{i}Q_{i}-M{\|}_{F}^{2}+\|Q_{i}{\|}_{2,1}+\mathrm{Tr}\left(A_{i}^{T}L_{m_{i}}A_{i}\right)\right)\\ & \;+\sum_{j=1}^{\bar{m}}\beta_{j}\left(\|S_{dj}-B_{j}B_{j}^{T}{\|}_{F}^{2}+\|B_{j}P_{j}-D{\|}_{F}^{2}+\|P_{j}{\|}_{2,1}+\mathrm{Tr}\left(B_{j}^{T}L_{d_{j}}B_{j}\right)\right)\\ & \;+\lambda \left(\|M{\|}_{F}^{2}+\|D{\|}_{F}^{2}+\|T{\|}_{F}^{2}\right)-{\left(1-k\right)}^{2}W, \end{array}$$ (22)

where *α*_*i*_ and *β*_*j*_ control the impact of auxiliary information. $A=\left\{A_{1},A_{2},\ldots ,A_{\bar{n}}\right\}$ and $B=\left\{B_{1},B_{2},\ldots ,B_{\bar{m}}\right\}$ are the potential representation matrices of miRNAs and diseases similarity; $Q=\left\{Q_{1},Q_{2},\ldots ,Q_{\bar{n}}\right\}$ and $P=\left\{P_{1},P_{2},\ldots ,P_{\bar{m}}\right\}$ are both projection matrices.

#### 3.3.4 WeightTDAIGN: Optimization Algorithm

Because the previous objective function in Eq. 22 is non-convex and the variables of the objective function are interdependent, we simplify the optimization problem by using the variable splitting technique. Finally, the optimization problem of Eq. 22 without updating the weight tensor W can be reformulated as follows:

$$\begin{array}{ll} & \underset{M,D,T,A,C,F,B,E,Q,P,G}{\min}\Phi =\|\sqrt{W}⊛\left(X-\left[\left[M,D,T\right]\right]\right){\|}_{F}^{2}\\ & +\sum_{i=1}^{\bar{n}}\alpha_{i}\left(\|S_{mi}-C_{i}A_{i}^{T}{\|}_{F}^{2}+\|A_{i}Q_{i}-M{\|}_{F}^{2}+\|Q_{i}{\|}_{2,1}+\mathrm{Tr}\left(F_{i}^{T}L_{m_{i}}F_{i}\right)\right)\\ & +\sum_{j=1}^{\bar{m}}\beta_{j}\left(\|S_{dj}-E_{j}B_{j}^{T}{\|}_{F}^{2}+\|B_{j}P_{j}-D{\|}_{F}^{2}+\|P_{j}{\|}_{2,1}+\mathrm{Tr}\left(G_{j}^{T}L_{d_{j}}G_{j}\right)\right)\\ & +\lambda \left(\|M{\|}_{F}^{2}+\|D{\|}_{F}^{2}+\|T{\|}_{F}^{2}\right)\\ & s.t\;C=A,E=B,F=A,G=B, \end{array}$$ (23)

where $C=\left\{C_{1},C_{2},\ldots ,C_{\bar{n}}\right\}$ , $F=\left\{F_{1},F_{2},\ldots ,F_{\bar{n}}\right\}$ , $E=\left\{E_{1},E_{2},\ldots ,E_{\bar{m}}\right\}$, and $G=\left\{G_{1},G_{2},\ldots ,G_{\bar{m}}\right\}$ are auxiliary variables.

By integrating the equality constraints into the objective function, we can construct the augmented Lagrangian function of Eq. 23 as follows:

$$\begin{array}{ll} L= & \Phi +\sum_{i=1}^{\bar{n}}\left(Y_{i}^{T}\left(C_{i}-A_{i}\right)+\frac{\mu }{2}\|C_{i}-A_{i}{\|}_{F}^{2}\right)\\ & +\sum_{i=1}^{\bar{n}}\left(H_{i}^{T}\left(F_{i}-A_{i}\right)+\frac{\gamma }{2}\|F_{i}-A_{i}{\|}_{F}^{2}\right)\\ & +\sum_{j=1}^{\bar{m}}\left(Z_{j}^{T}\left(E_{j}-B_{j}\right)+\frac{\eta }{2}\|E_{j}-B_{j}{\|}_{F}^{2}\right)\\ & +\sum_{j=1}^{\bar{m}}\left(J_{j}^{T}\left(G_{j}-B_{j}\right)+\frac{\varepsilon }{2}\|G_{j}-B_{j}{\|}_{F}^{2}\right), \end{array}$$ (24)

where $Y=\left\{Y_{1},Y_{2},\ldots ,Y_{\bar{n}}\right\}$ , $H=\left\{H_{1},H_{2},\ldots ,H_{\bar{n}}\right\}$ , $Z=\left\{Z_{1},Z_{2},\ldots ,Z_{\bar{m}}\right\}$, and $J=\left\{J_{1},J_{2},\ldots ,J_{\bar{m}}\right\}$ are the Lagrange multipliers. *μ*, *γ*, *η*, and *ɛ* are the penalty parameters.

Next, we develop an alternately updating rule and adopt the ADMM algorithm to optimize the objective function of Eq. 24.

Updating the factor matrices *M*, *D*, and *T*.

According to the idea of alternate iterative update, when the other variables are fixed, the terms in the objective function involving *M*, *D,* and *T* can be updated separately as follows:

$$\left\{\begin{array}{c} \underset{M}{\min}\|\sqrt{W_{\left(1\right)}}⊛\left(X_{\left(1\right)}-M{\left(D⊙T\right)}^{T}\right){\|}_{F}^{2}\;\\ +\overset{\bar{n}}{\underset{i=1}{\sum }}\alpha_{i}\|A_{i}Q_{i}-M{\|}_{F}^{2}+\lambda \|M{\|}_{F}^{2},\;\\ \underset{D}{\min}\|\sqrt{W_{\left(2\right)}}⊛\left(X_{\left(2\right)}-D{\left(M⊙T\right)}^{T}\right){\|}_{F}^{2}\;\\ +\overset{\bar{m}}{\underset{j=1}{\sum }}\beta_{j}\|B_{j}P_{j}-D{\|}_{F}^{2}+\lambda \|D{\|}_{F}^{2},\;\\ \underset{T}{\min}\|\sqrt{W_{\left(3\right)}}⊛\left(X_{\left(3\right)}-T{\left(M⊙D\right)}^{T}\right){\|}_{F}^{2}+\lambda \|T{\|}_{F}^{2},\; \end{array}\right.$$ (25)

where $X_{\left(1\right)}$ , $X_{\left(2\right)}$, and $X_{\left(3\right)}$ are the mode-1, mode-2, and mode-3 matricization of tensor X , respectively. ⊙ denotes the Khatri-Rao product. Similarly, $W_{\left(1\right)}$ , $W_{\left(2\right)}$, and $W_{\left(3\right)}$ are the mode-1, mode-2, and mode-3 matricization of tensor W , respectively.

Since the element-wise product of the tensor is involved in Eq. 25, we solve objective functions in Eq. 25 from the vector level. By letting the derivative of objective functions to zero, we can obtain the updating rules of *M*, *D,* and *T* as shown below:

$$\left\{\begin{array}{cc} M_{i,:}=\frac{{\left(W_{\left(1\right)}⊛X_{\left(1\right)}\left(D⊙T\right)+\sum_{i=1}^{\bar{n}}\alpha_{i}A_{i}Q_{i}\right)}_{i,:}}{{\left(D⊙T\right)}^{T}Diag\left(W_{\left(1\right)i,:}\right)\left(D⊙T\right)+\sum_{i=1}^{\bar{n}}\alpha_{i}I+\lambda I}, & \\ D_{i,:}=\frac{{\left(W_{\left(2\right)}⊛X_{\left(2\right)}\left(M⊙T\right)+\sum_{j=1}^{\bar{m}}\beta_{j}B_{j}P_{j}\right)}_{i,:}}{{\left(M⊙T\right)}^{T}Diag\left(W_{\left(2\right)i,:}\right)\left(M⊙T\right)+\sum_{j=1}^{\bar{m}}\beta_{j}I+\lambda I}, & \\ T_{i,:}=\frac{{\left(W_{\left(3\right)}⊛X_{\left(3\right)}\left(M⊙D\right)\right)}_{i,:}}{{\left(M⊙D\right)}^{T}Diag\left(W_{\left(3\right)i,:}\right)\left(M⊙D\right)+\lambda I}, \end{array}\right.$$ (26)

where $Diag\left(W_{\left(1\right)i,:}\right)$ , $Diag\left(W_{\left(2\right)i,:}\right)$, and $Diag\left(W_{\left(3\right)i,:}\right)$ refer to the diagonal matrices for the *i*th row vector of $W_{\left(1\right)}$ , $W_{\left(2\right)}$, and $W_{\left(3\right)}$ , respectively.

Updating the latent matrices *A*_*i*_, *C*_*i*_, *F*_*i*_, *B*_*j*_, *E*_*j*_, and *G*_*j*._

Similarly, we can solve other variables using the same solution strategy:

$$\left\{\begin{array}{cc} A_{i}=\frac{2\alpha_{i}S_{mi}^{T}C_{i}+2\alpha_{i}MQ_{i}^{T}+\mu C_{i}+Y_{i}+H_{i}+\gamma F_{i}}{2\alpha_{i}C_{i}^{T}C_{i}+2\alpha_{i}Q_{i}Q_{i}^{T}+\mu I+\gamma I}, & \\ C_{i}=\frac{2\alpha_{i}S_{mi}A_{i}+\mu A_{i}-Y_{i}}{2\alpha_{i}A_{i}^{T}A_{i}+\mu I}, & \\ F_{i}={\left(2\alpha_{i}L_{m_{i}}+\gamma I\right)}^{-1}\left(\gamma A_{i}-H_{i}\right), \end{array}\right.$$ (27)

$$\left\{\begin{array}{cc} B_{j}=\frac{2\beta_{j}S_{dj}^{T}E_{j}+2\beta_{j}DP_{j}^{T}+\eta E_{j}+Z_{j}+J_{j}+\varepsilon G_{j}}{2\beta_{j}E_{j}^{T}E_{j}+2\beta_{j}P_{j}P_{j}^{T}+\eta I+\varepsilon I}, & \\ E_{j}=\frac{2\beta_{j}S_{dj}B_{j}+\eta B_{j}-Z_{j}}{2\beta_{j}B_{j}^{T}B_{j}+\eta I}, & \\ G_{j}={\left(2\beta_{j}L_{d_{j}}+\varepsilon I\right)}^{-1}\left(\varepsilon B_{j}-J_{j}\right), \end{array}\right.$$ (28)

Updating the projection matrices *Q*_*i*_ and *P*_*j*._

According to previous research (Nie et al., 2010), the updating rules for projection matrices *Q*_*i*_ and *P*_*j*_ constrained by the *L*_2,1_ norm are as follows:

$$\left\{\begin{array}{cc} Q_{i}=\frac{M^{T}A_{i}}{A_{i}^{T}A_{i}+\frac{1}{2}\Lambda_{i}}, & \\ P_{j}=\frac{D^{T}B_{j}}{B_{j}^{T}B_{j}+\frac{1}{2}\sum_{j}}, \end{array}\right.$$ (29)

where $\Lambda_{i}\left(s,s\right)=\frac{1}{\left\|Q_{i}\left(s,:\right)\right\|}$ and $\sum_{j}\left(s,s\right)=\frac{1}{\left\|P_{j}\left(s,:\right)\right\|}$ are the diagonal matrix, and *Q*_*i*_ (*s*,:) and *P*_*j*_ (*s*,:) refer to the *s*th row vector of matrix *Q*_*i*_ and *P*_*j*_, respectively.

Updating the Lagrange multipliers *Y*_*i*_, *H*_*i*_, *Z*_*j*_, and *J*_*j*._

The updating formulations of Lagrange multipliers using gradient ascent can be defined as follows:

$$\left\{\begin{array}{cc} Y_{i}=Y_{i}+\mu \left(C_{i}-U_{i}\right), & \\ H_{i}=H_{i}+\gamma \left(F_{i}-U_{i}\right), & \\ Z_{j}=Z_{j}+\eta \left(E_{j}-V_{j}\right), & \\ J_{j}=J_{j}+\varepsilon \left(G_{j}-V_{j}\right), \end{array}\right.$$ (30)

Updating the weight tensor W.

In this article, we call the training process that reaches the stopping condition as an *episode*. When the aforementioned parameters are updated in an *episode*, the model starts to update the weight tensor W . The weight tensor W can be obtained through solving the following objective function:

$$\underset{W}{\min}\|\sqrt{W}⊛\left(X-\left[\left[M,D,T\right]\right]\right){\|}_{F}^{2}-{\left(1-k\right)}^{2}W.$$ (31)

Clearly, when *M*, *D*, and *T* are fixed, the optimal $W_{{\left(n\right)}_{ij}}$ can be easily calculated by

$$W_{{\left(n\right)}_{ij}}=\left\{\begin{array}{cc} 1,\; & \;\mathrm{if}l_{ij}\leq {\left(1-k\right)}^{2}\\ w,\; & \;\mathrm{otherwise}, \end{array}\right.$$ (32)

where the loss function *l*_*ij*_ is $\|X_{\left(1\right)}-M{\left(D⊙T\right)}^{T}{\|}_{F}^{2}$ for factor matrix *M*; the loss function *l*_*ij*_ is $\|X_{\left(2\right)}-D{\left(M⊙T\right)}^{T}{\|}_{F}^{2}$ for factor matrix *D*; and the loss function *l*_*ij*_ is $\|X_{\left(3\right)}-T{\left(D⊙M\right)}^{T}{\|}_{F}^{2}$ for factor matrix *T*.

Optimization algorithm.

According to the aforementioned alternately updating rules and ADMM algorithm, the final solution process for solving the optimization problem (Eq. 22) is summarized in Algorithm 1.

Algorithm 1 Algorithm for Solving Problem (Eq. 22).

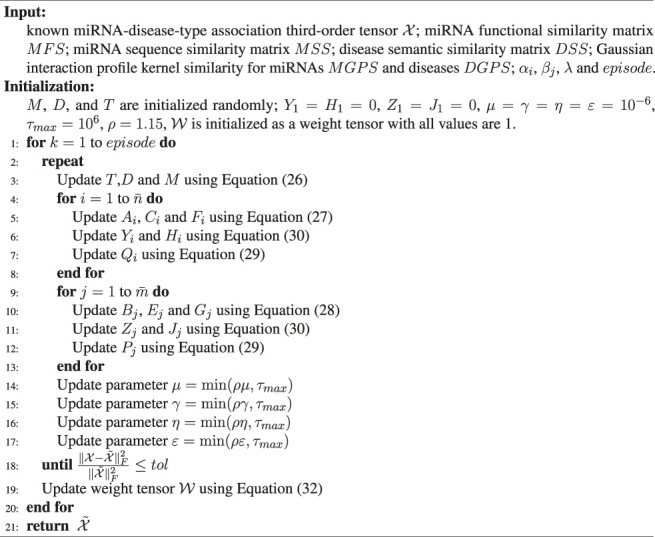

### 3.4 Complexity Analysis

We now analyze the time complexity of Algorithm 1 step by step as follows. In reality, we find that |*t*| is relatively small.

• **Updating the factor matrix** ***T*** **:** One must compute *M* ⊙ *D* first whose time complexity is O(|m‖n|r) . Then, the time complexity of updating $W_{\left(3\right)}⊛X_{\left(3\right)}\left(M⊙D\right)$ is O(|m‖n‖t|r) . Besides, the time complexity is $O\left(r{\left(\left|m\|n\right|\right)}^{2}+|m\|n|r^{2}\right)$ as we compute ${\left(M⊙D\right)}^{T}Diag\left(W_{\left(3\right)i,:}\right)\left(M⊙D\right)$ . Thus, the total time complexity of updating *T* is $O\left(r{\left(\left|m\|n\right|\right)}^{2}+|m\|n|r^{2}+|m\|n\|t|r\right)$ .
• **Updating the factor matrices** ***M*** **and** ***D*** **:** Similar to the previous steps, the time complexities of updating *M* and *D* are $O\left(r{\left(\left|n\|t\right|\right)}^{2}+|n\|t|r^{2}+|m\|n\|t|r\right)$ and $O\left(r{\left(\left|m\|t\right|\right)}^{2}+|m\|t|r^{2}+|m\|n\|t|r\right)$ , respectively.
• **Updating the latent matrices** ***A***_***i***_ **and** ***B***_***j***_ **:** According to the calculation of matrix multiplication complexity, the time complexities of updating *A*_*i*_ and *B*_*j*_ are $O\left(|m{|}^{2}r^{\prime }+|m|{\left(r^{\prime }\right)}^{2}+|m|rr^{\prime }+r{\left(r^{\prime }\right)}^{2}\right)$ and $O\left(|n{|}^{2}r^{\prime }+|n|{\left(r^{\prime }\right)}^{2}+|n|rr^{\prime }+r{\left(r^{\prime }\right)}^{2}\right)$ , respectively.
• **Updating the latent matrices** ***C***_***i***_ **,** ***F***_***i***_ **,** ***E***_***j***,_ **and** ***G***_***j***_ **:** Similar to the complexities of computing *A*_*i*_ and *B*_*j*_, the time complexities of computing *C*_*i*_ and *E*_*j*_ are $O\left(|m{|}^{2}r^{\prime }+|m|{\left(r^{\prime }\right)}^{2}\right)$ and $O\left(|n{|}^{2}r^{\prime }+|n|{\left(r^{\prime }\right)}^{2}\right)$ , respectively. In addition, computing *F*_*i*_ and *G*_*j*_ need $O\left(|m{|}^{2}r^{\prime }\right)$ and $O\left(|n{|}^{2}r^{\prime }\right)$ time, respectively.
• **Updating the projection matrices** ***Q***_***i***_ **and** ***P***_***j***_ **:** One needs to compute the projection matrices *Q*_*i*_ and *P*_*j*_, whose time complexities are $O\left(|m|{\left(r^{\prime }\right)}^{2}+|m|rr^{\prime }+r{\left(r^{\prime }\right)}^{2}\right)$ and $O\left(|n|{\left(r^{\prime }\right)}^{2}+|n|rr^{\prime }+r{\left(r^{\prime }\right)}^{2}\right)$ , respectively.
• **Updating the weight tensor** W **:** For mode-*n* matricization $W_{\left(n\right)}$ , its time complexity is O(|m‖n‖t|) .

Finally, as the number of iterations is constant and the time complexity of updating the Lagrange multipliers is $O\left(|m|r^{\prime }\right)$ or $O\left(|n|r^{\prime }\right)$ , the total time complexity for each iteration of Algorithm 1 is $O\left(r{\left(\left|m\|n\right|\right)}^{2}+r{\left(\left|n\|t\right|\right)}^{2}+r{\left(\left|m\|t\right|\right)}^{2}+|m\|n\|t|r\right)$ , where *r* ≪ min (|*m*|, |*n*|) and *r*′ ≪ min (|*m*|, |*n*|).

## 4 Results

### 4.1 Implementation Details and Evaluation Metrics

In this article, our goal is to more accurately predict positive samples while discovering the potential multiple types of miRNA–disease associations. To evaluate the performance of models more comprehensively from this idea, two different cases are considered under 5-fold cross-validation.

• CV_*type*_: we randomly split all miRNA–disease pairs which include not less than one type of association into five equal-sized subsets. It is worth noting that we must ensure the five equal-sized subsets do not contain each other. In each fold, one subset is served as a testing set in turn, and the rest of the subsets as a training set. According to the predicted score, the prediction results of all association types are ranked for each miRNA–disease pair in the testing set. Then, we use the type with the highest score as the final prediction result for the test sample and calculate the Top-1 precision, Top-1 recall, and Top-1 F1.
• CV_*triplet*_: we divide the training set and testing set in the same way as CV_*type*_. In each turn, we mainly evaluate whether known miRNA–disease-type triplets are well predicted. To better evaluate the ability of models to predict positive samples, the area under the precision-recall (AUPR) curve, the area under the receiver operating characteristic (AUC) curve, and mean square error (MSE) are calculated to evaluate the prediction performance of all models.

Obviously, the problem of predicting the multiple-type associations between miRNAs and diseases is what we are more concerned about. Therefore, we regard CV_*type*_ as our primary experimental setting. Moreover, we use Python 3.8.5 and the tensor learning tool “tensorly” to implement comparative experiments between our proposed WeightTDAIGN and all benchmark models.

### 4.2 Parameters Analysis

**FIGURE 2:**
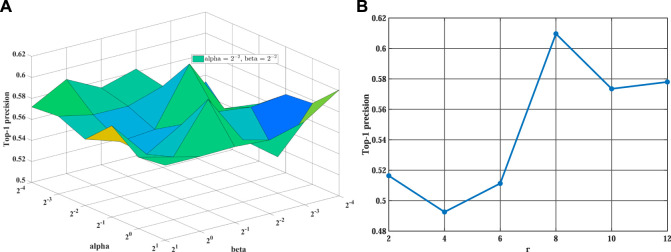
The influence of different hyperparameters on WeightTDAIGN based on the MDA v2.0–4 dataset. **(A)** The impact of hyperparameters *α* and *β* WeightTDAIGN and **(B)** the impact of hyperparameter *r* on WeightTDAIGN. Note that to facilitate visualization panel (A), we use 2^*n*^ to represent 2 × 10^*n*^ when *n* < 0.

**FIGURE 3:**
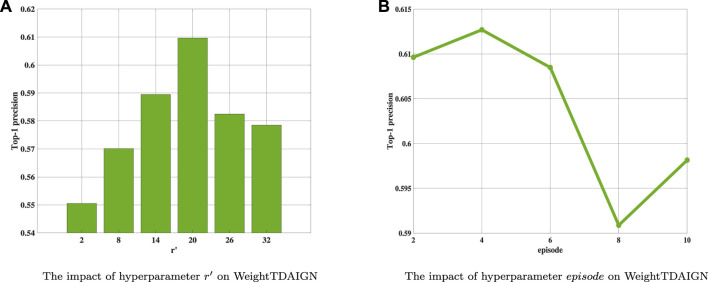
The influence of different hyperparameters on WeightTDAIGN based on the MDA v2.0–4 dataset. **(A)** The impact of hyperparameters *r*′ WeightTDAIGN and **(B)** the impact of hyperparameter *episode* on WeightTDAIGN.

**FIGURE 4:**
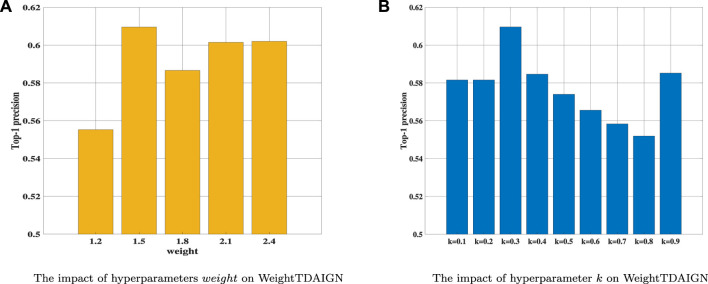
The influence of different hyperparameters on WeightTDAIGN based on the MDA v2.0–4 dataset. **(A)** The impact of hyperparameters *weight* WeightTDAIGN and **(B)** the impact of hyperparameter *k* on WeightTDAIGN.

Before the training stage, we apply grid search and 5-fold cross-validation to find the optimal hyperparameters on different sparsity datasets. We take the MDA v2.0–4 dataset as an example to show the process of cross-validation. First, we fix the value of *λ* at 0.001 and search for the optimal values of other parameters. Then, we find the optimal value of *α* and *β* from {2^–4^, 2^–3^, 2^–2^, 2^–1^, 2^0^, 2^1^} and set *α* = 2^–2^ and *β* = 2^–2^ in our experiment (see Figure 2A). Training tensor rank *r* within {2, 4, 6, 8, 10, 12} and setting *r* = 8 (see Figure 2B). Varying matrix rank *r*′ in the set {2, 8, 14, 20, 26, 32} and setting *r*′ = 20 in Figure 3A. Moreover, we find that setting different episodes also has a certain impact on the performance of the model. Therefore, we search the optimal *episode* from {2, 4, 6, 8, 10} and set *episode* as 4 (see Figure 3B). Obviously, when the value in the weight tensor W is larger, the hypothesis space is found by the model tends to increase the value of elements for the reconstructed tensor $\tilde{X}$ , which leads to too many false positives samples in the predicted value. Thus, to overcome the aforementioned problem, we utilize Top-1 precision as a metric to find the optimal weight and set the same weight for different slices. We can see that when the weight is set to 1.5, the model can achieve the best performance in Figure 4A. Finally, training *k* within {0.1, 0.2, 0.3, 0.4, 0.5, 0.6, 0.7, 0.8, 0.9} and setting *k* = 0.3 (see Figure 4B). It is worth noting that other experimental data in this article should also use the previous steps for hyperparameters selection. Moreover, the detailed parameter adjustment results of other data are available in the Supplementary Parameters Analysis.

### 4.3 Comparison Experiments

To compare the performance of our model more comprehensively, we introduce one representative prediction model RBMMMDA and three tensor decomposition models, CP decomposition (CP), tensor factorization using auxiliary information (TFAI) (Narita et al., 2012), and tensor decomposition with relational constraints (TDRC) as baselines. RBMMMDA is the first model to study the problem of multiple types of miRNA–disease associations. CP model is a standard tensor decomposition method without taking into account biological similarity information. TFAI only considers the inter-data connection relations in biological similarity matrices by introducing graph Laplacian regularization. TDRC only focuses on integrating biological similarity information into the CP decomposition via the strategy of alternately updating factor matrices and projection matrices. At the same time, in order to show that weighting positive samples, the introduction of *L*_2,1_ norm, and graph Laplacian regularization can effectively improve the prediction performance of the model, we propose the TDAIGN model without weight and the TDAI model without *L*_2,1_ norm and graph Laplacian regularization, respectively. For convenience, we call RBMMMDA, CP, TFAI, TDRC, TDAI, and TDAIGN models as benchmark models. To better demonstrate the optimal performance of benchmark models, we carry out 5-fold cross-validation for TDRC and TDAIGN models to select the optimal hyperparameters *α* and *β*. For fairness, we uniformly set the same rank as WeightTDAIGN for benchmark models on different sparsity datasets. Moreover, we use the same convergence criteria for CP, TFAI, TDRC, TDAI, and TDAIGN. Finally, the detailed parameter settings for all benchmark models can be found in Supplementary Comparison Methods for Parameter Analysis.

Due to the imbalance of positive and negative samples on the datasets, we randomly select the same number of unobserved elements and treat them as negative samples. In addition, we compare and analyze the results of different models under 5-fold cross-validation on MDA v2.0–2, MDA v2.0–3, MDA v2.0–4, and MDA v3.2–5 datasets. As shown in Table 2, we find that the WeightTDAIGN model achieves the highest AUC, AUPR, and F1 values compared with benchmark models on all datasets. Moreover, in terms of MSE used to measure the difference between the true value and the predicted value in positive samples, WeightTDAIGN is also significantly smaller than benchmark models on all datasets. The analysis of the previous results indicates that compared with the six benchmark models, WeightTDAIGN can recover positive samples well and improve prediction performance by weighting positive samples.

**TABLE 2:** The performance of all models evaluated by 5-fold cross-validation under CV_*triplet*_.

		AUC	AUPR	F1	MSE
MDA v2.0-2	RBMMMDA^a^	0.855252	0.845632	0.791036	0.819910
—	CP	0.912348	0.924856	0.834056	0.538964
—	TFAI	0.911866	0.924786	0.835192	0.540715
—	TDRC	0.900086	0.915792	0.822848	0.559964
—	TDAI	0.908280	0.920506	0.828144	0.549998
—	TDAIGN	0.910948	0.923750	0.829668	0.539089
—	WeightTDAIGN	**0.933064**	**0.941610**	**0.861922**	**0.484437**
MDA v2.0-3	RBMMMDA^a^	0.787792	0.777968	0.744724	0.734902
—	CP	0.944596	0.955340	0.875980	0.298760
—	TFAI	0.936446	0.947686	0.861838	0.357501
—	TDRC	0.931564	0.942962	0.852522	0.362024
—	TDAI	0.944558	0.955166	0.875612	0.308545
—	TDAIGN	0.948034	0.957388	0.876888	0.307712
—	WeightTDAIGN	**0.971160**	**0.974772**	**0.915732**	**0.258653**
MDA v2.0-4	RBMMMDA^a^	0.790984	0.781692	0.746280	0.674311
—	CP	0.930410	0.943304	0.852792	0.322797
—	TFAI	0.933182	0.944776	0.853160	0.323449
—	TDRC	0.928236	0.940798	0.852378	0.338039
—	TDAI	0.933482	0.944868	0.856652	0.323383
—	TDAIGN	0.933978	0.945050	0.856182	0.323505
—	WeightTDAIGN	**0.955112**	**0.960600**	**0.886136**	**0.283076**
MDA v3.2-5	RBMMMDA^a^	0.857432	0.852302	0.787936	0.568752
—	CP	0.862516	0.867546	0.789574	0.514538
—	TFAI	0.862770	0.867616	0.790276	0.514886
—	TDRC	0.861966	0.866272	0.790432	0.512712
—	TDAI	0.862536	0.867434	0.789696	0.514705
—	TDAIGN	0.862640	0.867566	0.789866	0.514937
—	WeightTDAIGN	**0.868180**	**0.869516**	**0.796142**	**0.472897**

a Since the open-source web server can no longer be used, the reported results here are our re-implementation of the original algorithms.

To comprehensively evaluate the performance of all models in predicting multiple types of miRNA–disease associations, we conduct experiments on different sparsity datasets under CV_*type*_. From Table 3 we can see that the prediction performance of WeightTDAIGN is better than that of the six benchmark models. To be more specific, WeightTDAIGN is significantly better than the latest TDRC model on MDA v2.0–2, MDA v2.0–3, and MDA v2.0–4 datasets. In particular, WeightTDAIGN obtains certain performance gains over TDRC by 8.40% in terms of Top-1 precision, 8.40% in terms of Top-1 recall, and 8.44% in terms of Top-1 F1 on sparsest MDA v2.0–2 dataset. Moreover, even for the relatively dense MDA v3.2–5 dataset, WeightTDAIGN still shows good prediction performance. These indicate that WeightTDAIGN has certain competitiveness, especially when the datasets are relatively sparse, in the task of predicting multiple types of miRNA–disease associations. In addition, we observe that the prediction performance of TDAIGN is better than TDAI on all datasets, which indicates that the introduction of graph Laplacian regularization and *L*_2,1_ norm can effectively preserve the local structural information of biological similarity networks and reduce the noise information contained in these networks. Furthermore, we find that the better prediction performance of WeightTDAIGN can be achieved compared with TDAIGN on all datasets. This shows that weighting positive samples can effectively improve the prediction performance of the model. Last but not least, compared with RBMMMDA, CP, and TFAI models, TDAIGN achieves better prediction performance on all datasets. This indicates that more similar information related to miRNAs and diseases is integrated into the model, which can effectively improve the prediction performance of the model and solve the problem of tensor sparseness.

**TABLE 3:** The performance of all models evaluated by 5-fold cross-validation under CV_*type*_.

		Top-1 precision	Top-1 recall	Top-1 F1
MDA v2.0-2	RBMMMDA^a^	0.368033	0.318645	0.323771
—	CP	0.584426	0.505831	0.528142
—	TFAI	0.587705	0.508327	0.526776
—	TDRC	0.585246	0.506537	0.529235
—	TDAI	0.575410	0.498121	0.524454
—	TDAIGN	0.609016	0.527208	0.550273
—	WeightTDAIGN	**0.634426**	**0.549107**	**0.573907**
MDA v2.0-3	RBMMMDA^a^	0.390437	0.309279	0.318581
—	CP	0.568611	0.452077	0.492431
—	TFAI	0.583551	0.463528	0.492374
—	TDRC	0.585770	0.466194	0.495993
—	TDAI	0.572821	0.454553	0.492710
—	TDAIGN	0.585747	0.465819	0.500618
—	WeightTDAIGN	**0.607207**	**0.482831**	**0.512076**
MDA v2.0-4	RBMMMDA^a^	0.359857	0.269427	0.277623
—	CP	0.544385	0.409100	0.433500
—	TFAI	0.555865	0.416682	0.443161
—	TDRC	0.577683	0.434773	0.467879
—	TDAI	0.569554	0.425020	0.454890
—	TDAIGN	0.577398	0.431664	0.462733
—	WeightTDAIGN	**0.620535**	**0.465670**	**0.496494**
MDA v3.2-5	RBMMMDA^a^	0.542408	0.325892	0.347250
—	CP	0.581700	0.349530	0.377299
—	TFAI	0.585528	0.351849	0.381735
—	TDRC	0.597008	0.358833	0.388722
—	TDAI	0.580311	0.348687	0.375127
—	TDAIGN	0.588309	0.353523	0.383068
—	WeightTDAIGN	**0.606737**	**0.364620**	**0.398307**

a Since the open-source web server can no longer be used, the reported results here are our re-implementation of the original algorithms.

### 4.4 Case Studies

**FIGURE 5:**
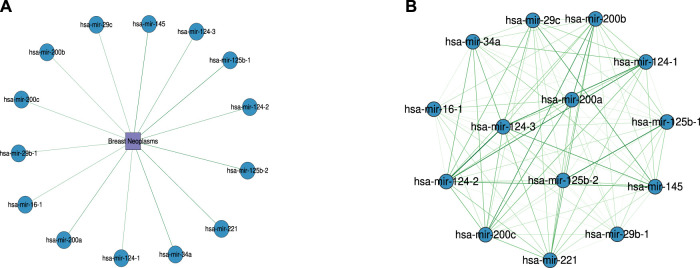
The association network of the top 50 predictions for miRNAs with type as the target in breast neoplasms. **(A)** Predicted association between miRNAs and breast neoplasms. **(B)** Functional similarity network between miRNAs associated with breast neoplasms. Darker colors indicate higher similarity between miRNAs. The similarity values range from 0.5 to 1.

To further evaluate the ability of WeightTDAIGN to predict the potential multiple-type associations between miRNAs and diseases, we build the model by using all four known types of miRNA–disease associations on the HMDD v2.0 dataset and then predict those unknown miRNA–disease-type triplets. Further, we verify the prediction results on the HMDD v3.2 dataset and recent literature. Moreover, in order to comprehensively demonstrate the generalization performance of WeihghtTDAIGN on different sparsity datasets, we conduct case studies on MDA v2.0–2, MDA v2.0–3, and MDA v2.0–4 datasets, respectively. The higher the predictive scores for unobserved miRNA–disease-type triplets, the higher the probability of correct prediction, so we only verify the results using the top predictive scores. The results are shown in Table 4. We can see that all of the top 50 disease-related miRNAs are successfully confirmed by HMDD v3.2 for MDA v2.0–4 dataset. Meanwhile, Supplementary Table S2 shows that 99 of the top 100 are verified by HMDD v3.2 for MDA v2.0–2 dataset. Supplementary Table S3 demonstrates the top 100 predicted results and ninety-seven predictions can be confirmed according to recent literature for MDA v2.0–3 dataset. We also find that miRNAs (or diseases) with very high similarities are predicted to be associated with the same disease (or miRNA), and these miRNAs belong to the same type. For example, in terms of MDA v2.0–4 dataset, hsa-mir-124-1, hsa-mir-124-2, and hsa-mir-124-3 suppress multiple steps of breast cancer metastasis by targeting a cohort of pro-metastatic genes *in vitro* (Lv et al., 2011). Further, Figure 5 presents the similarity network of miRNAs associated with breast neoplasms in the top 50 association predictions. Clearly, there is a high functional similarity between miRNAs of the same type that are highly associated with breast neoplasms. This further shows that the necessity of incorporating more biological similarity networks and using graph Laplacian regularization can well capture the internal node similarity relations between miRNAs and between diseases.

**TABLE 4:** Top 50 disease-related miRNAs predicted by WeightTDAIGN based on MDA v2.0–4.

miRNA	Disease	Type	Score	PMID	miRNA	Disease	Type	Score	PMID
hsa-mir-34a	Colorectal neoplasms	Target	1.424456	24370784	hsa-mir-200b	Prostatic neoplasms	Target	1.028036	21224847
hsa-mir-17	Carcinoma and hepatocellular	Target	1.325876	23418359	hsa-mir-124-1	Breast neoplasms	Target	1.003782	22085528
hsa-mir-145	Breast neoplasms	Target	1.307542	19360360	hsa-mir-19a	Carcinoma and hepatocellular	Target	0.985647	28724429
hsa-mir-125b-1	Carcinoma and hepatocellular	Target	1.289963	22293115	hsa-mir-17	Melanoma	Circulation	0.983089	20529253
hsa-mir-125b-1	Breast neoplasms	Target	1.267228	19738052	hsa-mir-19b-1	Carcinoma and hepatocellular	Target	0.975428	17188425
hsa-mir-15a	Leukemia, lymphocytic, chronic, and B cell	Target	1.258953	19498445	hsa-mir-200c	Carcinoma and hepatocellular	Epigenetics	0.974930	23222811
hsa-mir-125b-2	Breast neoplasms	Target	1.230360	19738052	hsa-mir-16-1	Multiple myeloma	Target	0.967225	23104180
hsa-mir-34a	Breast neoplasms	Target	1.227899	21814748	hsa-mir-18a	Breast neoplasms	Genetics	0.957834	16754881
hsa-mir-16-1	Leukemia, lymphocytic, chronic, and B cell	Target	1.199684	19498445	hsa-mir-29c	Breast neoplasms	Target	0.956380	22330642
hsa-mir-125b-2	Carcinoma and hepatocellular	Target	1.166586	22293115	hsa-mir-200c	Breast neoplasms	Target	0.948673	23209748
hsa-mir-200b	Carcinoma and hepatocellular	Epigenetics	1.162828	22370893	hsa-mir-29b-1	Breast neoplasms	Target	0.939779	22864815
hsa-mir-221	Breast neoplasms	Target	1.152046	21868360	hsa-mir-15a	Multiple myeloma	Target	0.937730	23104180
hsa-mir-19b-1	Breast neoplasms	Genetics	1.130424	16754881	hsa-mir-16-1	Breast neoplasms	Target	0.933405	19250063
hsa-mir-200a	Carcinoma and hepatocellular	Epigenetics	1.127118	21837748	hsa-mir-200a	Prostatic neoplasms	Target	0.929946	21224847
hsa-mir-200a	Breast neoplasms	Target	1.118853	21926171	hsa-mir-18a	Carcinoma and hepatocellular	Genetics	0.925369	15944709
hsa-mir-124-2	Carcinoma and hepatocellular	Target	1.110440	21672940	hsa-mir-17	Colorectal neoplasms	Epigenetics	0.924489	22308110
hsa-mir-124-3	Carcinoma and hepatocellular	Target	1.110440	21672940	hsa-mir-16-1	Prostatic neoplasms	Genetics	0.922385	17940623
hsa-mir-19a	Breast neoplasms	Genetics	1.079414	16754881	hsa-mir-16-1	Carcinoma and hepatocellular	Target	0.916582	23226427
hsa-mir-124-2	Breast neoplasms	Target	1.078512	22085528	hsa-mir-218-1	Breast neoplasms	Genetics	0.913360	16754881
hsa-mir-124-3	Breast neoplasms	Target	1.078512	22085528	hsa-mir-124-1	Carcinoma and hepatocellular	Target	0.912254	21672940
hsa-mir-200c	Stomach neoplasms	Target	1.057460	25986864	hsa-mir-16-2	Carcinoma and hepatocellular	Target	0.910880	23226427
hsa-mir-200c	Prostatic neoplasms	Target	1.054738	21224847	hsa-mir-15a	Prostatic neoplasms	Genetics	0.906294	17940623
hsa-mir-17	Breast neoplasms	Genetics	1.049201	16754881	hsa-mir-133a-2	Colorectal neoplasms	Epigenetics	0.906253	22766685
hsa-mir-126	Carcinoma and non-small cell lung	Circulation	1.046761	22009180	hsa-mir-34a	Prostatic neoplasms	Target	0.892918	21240262
hsa-mir-31	Breast neoplasms	Epigenetics	1.045268	22289355	hsa-mir-200b	Breast neoplasms	Target	0.889298	20514023

**FIGURE 6:**
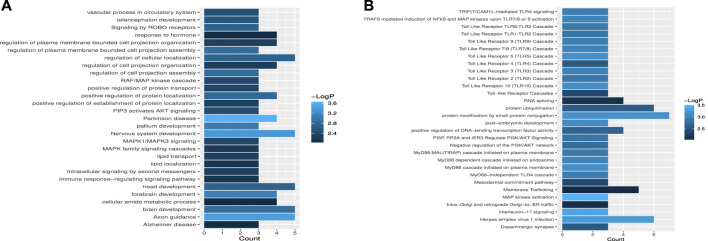
The enrichment analysis of miRNA target gene sets. **(A)** The statistical significance of target gene sets associated with hsa-mir-218-1. **(B)** The statistical significance of target gene sets associated with hsa-mir-218-2.

To further validate the biological significance of the potential miRNA–disease associations discovered by the WeightTDAIGN model, we perform enrichment analysis for gene sets composed of miRNA target genes. The target genes of each miRNA are obtained from miRTarBase (Huang et al., 2020), and Metascape (Zhou et al., 2019) is used to explore whether the obtained target gene sets are related to important pathways or receptor regulating diseases. From Figure 6A, we can see that among the target genes related to hsa-mir-218-1, some target genes are associated with signaling pathways such as MAPK1/MAPK3 signaling, MAPK family signaling cascades and PIP3 activates AKT signaling. Also, many studies have reported that the expression of MAPK is closely related to tumor invasion and metastasis in breast neoplasms, and the activation of AKT signaling will promote tumor initiation and progression (Jiang et al., 2020; He et al., 2021). Similarly, some target genes of hsa-mir-218-2 are associated with toll-like receptor in Figure 6B, while toll-like receptor has an important association with the occurrence and development of breast neoplasms (Shi et al., 2020). In conclusion, hsa-mir-218-1 and hsa-mir-218-2 may be closely related to the occurrence and development of breast neoplasms.

## 5 Discussion

While predicting the associations between miRNAs and diseases, we can also determine the specific type of miRNA, which is very helpful for a more detailed understanding of the pathogenesis of the disease at the molecular level. In this article, we propose the WeightTDAIGN model based on CP decomposition by introducing weight, graph Laplacian regularization, and *L*_2,1_ norm, which also incorporates more auxiliary information into the framework of tensor decomposition. Experimental results demonstrate that WeightTDAIGN can recover positive samples well compared with six benchmark models under CV_*triplet*_. Meanwhile, WeightTDAIGN can also achieve satisfactory prediction performance on different sparsity datasets under CV_*type*_. All of the above show that WeightTDAIGN our proposed can effectively improve the performance and robustness of predicting the associations of multiple types of miRNA–disease. In addition, the comparative experiment of TDAI and TDAIGN indicates that the introduction of graph Laplacian regularization and *L*_2,1_ norm contributes to preserving the local structure information of similarity networks and reducing the influence of noise in these networks on the model. Also, the comparative experiment of TDAIGN and WeightTDAIGN confirms that weighting positive samples can recover positive samples well and improve the prediction performance of the model. Moreover, the results of the case studies further demonstrate that WeightTDAIGN can accurately predict the associations of multiple types of miRNA–disease and discover the potential associations of unconfirmed miRNA–disease that are of biological significance in enrichment analysis. In conclusion, WeightTDAIGN can serve as a powerful tool to infer the multiple-type associations between miRNAs and diseases rather than simply predicting disease-related miRNAs.

## Data Availability

The original contributions presented in the study are included in the article/Supplementary Material; further inquiries can be directed to the corresponding author.
