# Corrigendum: Predicting multiple types of associations between miRNAs and diseases based on graph regularized weighted tensor decomposition

**DOI:** 10.3389/fbioe.2022.1006237

**Published:** 2022-08-30

**Authors:** Dong Ouyang, Rui Miao, Jianjun Wang, Xiaoying Liu, Shengli Xie, Ning Ai, Qi Dang, Yong Liang

**Affiliations:** ^1^ Faculty of Information Technology, Macau University of Science and Technology, Macau, China; ^2^ School of Mathematics and Statistics, Southwest University, Chongqing, China; ^3^ Computer Engineering Technical College, Guangdong Polytechnic of Science and Technology, Zhuhai, China; ^4^ Institute of Intelligent Information Processing, Guangdong University of Technology, Guangzhou, China; ^5^ Peng Cheng Laboratory, Shenzhen, China

**Keywords:** multiple types of miRNA–disease associations, weighted tensor decomposition, graph Laplacian regularization, L2, 1 norm, multi-view biological similarity network

In the published article, there was an error in affiliation(s) **1**. Instead of “Faculty of Information Technology, Macau University of Science and Technology, Taipa, China,” it should be “Faculty of Information Technology, Macau University of Science and Technology, Macau, China.”

In the published article, there was an error. **Mathematical symbols are inconsistent**.

A correction has been made to **3 Methods**, “*3.1 CP decomposition*,” Paragraph Number 5.

This sentence previously stated:

“CANDECOMP/PARAFAC (CP) decomposition is one of the most common tensor decomposition forms (Kolda and Bader, 2009). Given the miRNA-disease-type tensor $X\in R^{\left|m|\times |n|\times |t\right|}$ , the CP decomposition model can be represented as follows:

$$X\approx \overset{S}{\underset{s=1}{\sum }}m_{s}◦d_{s}◦t_{s}\equiv \left[\left[M,D,T\right]\right]$$ (1)

where the symbol ◦ represents the vector outer product, *S* is a positive integer and $m_{s}\in R^{\left|m|\times |1\right|}$ , $d_{s}\in R^{\left|n|\times |1\right|}$ and $t_{s}\in R^{\left|t|\times |1\right|}$ . *M* = [***m***_1_ ***m***_2_ ⋯ ***m***_*S*_], *D* = [***d***_1_ ***d***_2_ ⋯ ***d***_*S*_], and *T* = [***t***_1_ ***t***_2_ ⋯ ***t***_*S*_] are the factor matrices with respect to different dimensions.”

The corrected sentence appears below:

“CANDECOMP/PARAFAC (CP) decomposition is one of the most common tensor decomposition forms (Kolda and Bader, 2009). Given the miRNA-disease-type tensor $X\in R^{\left|m|\times |n|\times |t\right|}$ , the CP decomposition model can be represented as follows:

$$X\approx \overset{S}{\underset{s=1}{\sum }}m_{s}◦d_{s}◦t_{s}\equiv \left[\left[M,D,T\right]\right]$$ (2)

where the symbol ◦ represents the vector outer product, *S* is a positive integer and $m_{s}\in R^{|m|\times 1}$ , $d_{s}\in R^{|n|\times 1}$ and $t_{s}\in R^{|t|\times 1}$ . *M* = [***m***_1_ ***m***_2_ ⋯ ***m***_*S*_], *D* = [***d***_1_ ***d***_2_ ⋯ ***d***_*S*_], and *T* = [***t***_1_ ***t***_2_ ⋯ ***t***_*S*_] are the factor matrices with respect to different dimensions.”

Note that mathematic symbols are bolded to represent vectors. Also, “ $m_{s}\in R^{\left|m|\times |1\right|}$ , $d_{s}\in R^{\left|n|\times |1\right|}$ and $t_{s}\in R^{\left|t|\times |1\right|}$ ” should be changed to “ $m_{s}\in R^{|m|\times 1}$ , $d_{s}\in R^{|n|\times 1}$ and $t_{s}\in R^{|t|\times 1}$ .”

The authors apologize for this error and state that this does not change the scientific conclusions of the article in any way. The original article has been updated.

## References

[B1] Kolda T. G. Bader B. W. (2009). Tensor decompositions and applications. SIAM Rev. 51, 455–500. 10.1137/07070111X

