# Supplementary material for: Predicting Multiple Types of Associations Between miRNAs and Diseases Based on Graph Regularized Weighted Tensor Decomposition
Source: Front Bioeng Biotechnol. 2022 Jul 4;10:911769. doi: 10.3389/fbioe.2022.911769 (PMC9335924; doi:10.3389/fbioe.2022.911769)
Supplement: Supplementary file 1 [file DataSheet1.docx]

Supplementary Material

# Supplementary Parameters Analysis

To show the best performance of the WeightTDAIGN model more fairly and reasonably, we perform 5-fold cross validation on the MDAv2.0-2, MDAv2.0-3 and MDAv3.2-5 datasets to select the optimal parameters. The parameter adjustment process is as follows:

For the MDAv2.0-2 dataset, we adjust the seven hyperparameters as shown in Supplementary Figure 1:


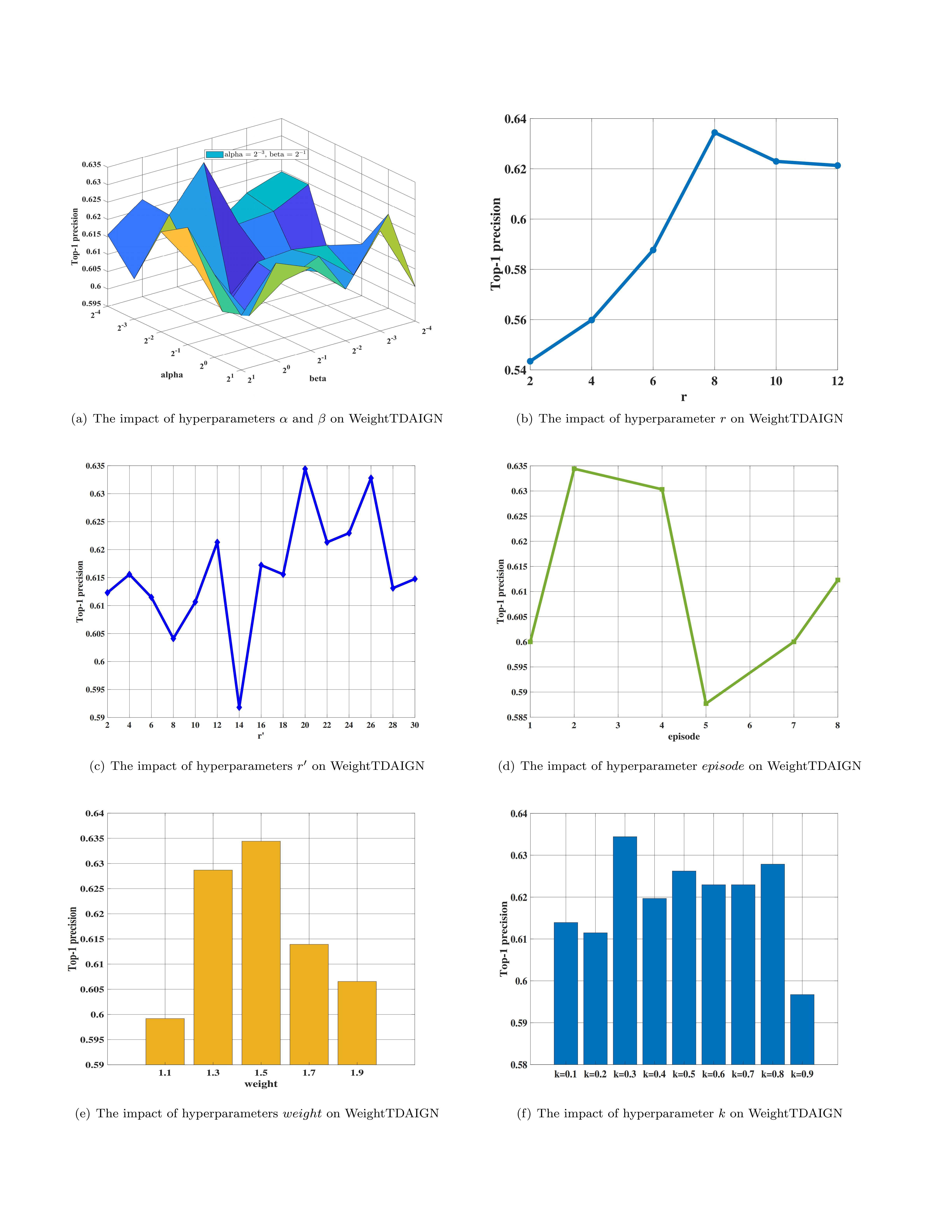


**Supplementary Figure 1.** The influence of different hyperparameters on WeightTDAIGN based on MDAv2.0-2 dataset.

For the MDAv2.0-3 dataset, we adjust the seven hyperparameters as shown in Supplementary Figure 2:


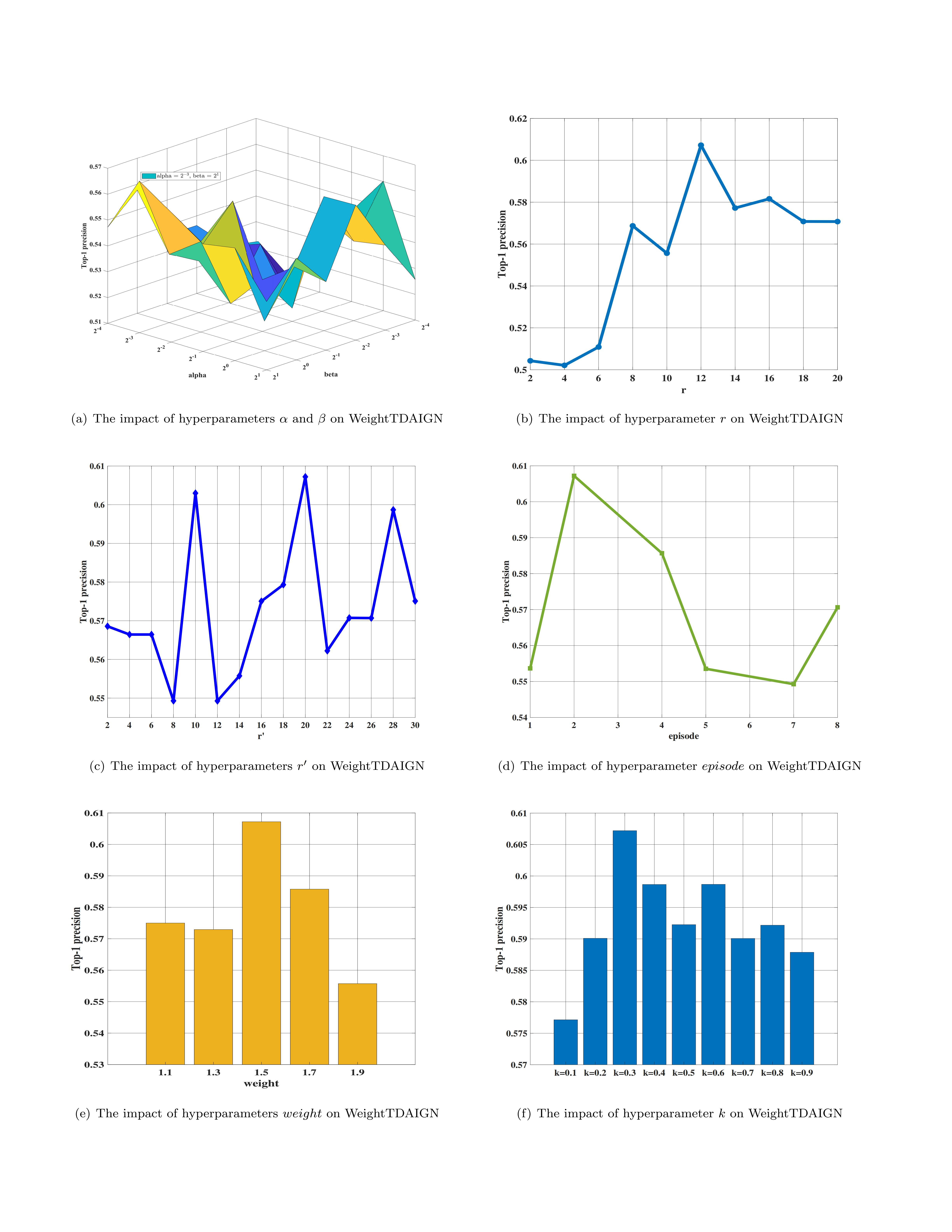


**Supplementary Figure 2.** The influence of different hyperparameters on WeightTDAIGN based on MDAv2.0-3 dataset.

For the MDAv3.2-5 dataset, we adjust the seven hyperparameters as shown in Supplementary Figure 3:


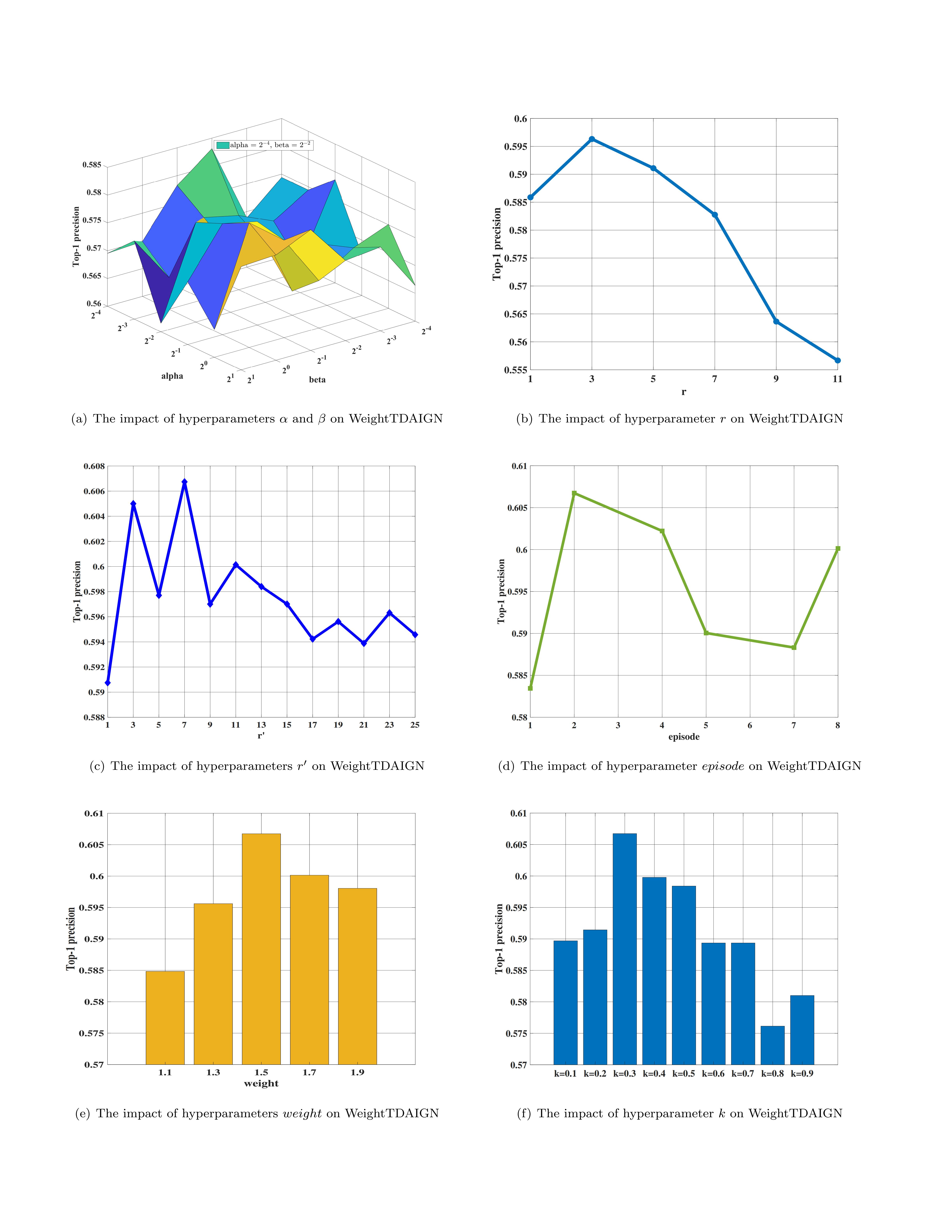


**Supplementary Figure 3.** The influence of different hyperparameters on WeightTDAIGN based on MDAv3.2-5 dataset.

# Supplementary Comparison Methods for Parameters Analysis

Supplementary Table 1 shows the details of hyperparameter settings for benchmark models. Compared with the other hyperparameters, $\lambda$ has less influence on performance. We empirically set $\lambda$ = 0.001. In addition, we uniformly set the same rank as WeightTDAIGN for benchmark models on different sparsity datasets for fairness. To better demonstrate the optimal performance of benchmark models, we carry out 5-fold cross validation for TDRC and TDAIGN models to select the optimal hyperparameters $\alpha$ and $\beta$. The detailed parameter tuning results on different datasets are shown in Supplementary Figure 4 and Supplementary Figure 5. Based on previous research [1], the Laplacian regularization coefficients also control the contributions of miRNA-miRNA similarity and disease-disease similarity in TFAI, so we set $\alpha$ and $\beta$ as the same as the settings in TDRC on different datasets. Similarly, the hyperparameters $\alpha$ and $\beta$ in TDAI are also set the same as TDAIGN on different datasets. It is worth noting that all of our comparison models are implemented using python 3.8.5, tensor learning tool "tensorly" (version 0.5.1), numpy (version 1.21.5) and pandas (version 1.3.5).

**Supplementary Table 1.** The hyperparameter settings of benchmark models.

|  | hyperparameters | CP | TFAI | TDRC | TDAI | TDAIGN |
| --- | --- | --- | --- | --- | --- | --- |
| MDAv2.0-2 | $r$ | 8 | 8 | 8 | 8 | 8 |
|  | $r^{'}$ | $\times$ | $\times$ | $\times$ | 20 | 20 |
|  | $\alpha$ | $\times$ | $2^{-3}$ | $2^{-3}$ | $2^{0}$ | $2^{0}$ |
|  | $\beta$ | $\times$ | $2^{0}$ | $2^{0}$ | $2^{-3}$ | $2^{-3}$ |
|  | $\lambda$ | 0.001 | 0.001 | 0.001 | 0.001 | 0.001 |
| MDAv2.0-3 | $r$ | 12 | 12 | 12 | 12 | 12 |
|  | $r^{'}$ | $\times$ | $\times$ | $\times$ | 20 | 20 |
|  | $\alpha$ | $\times$ | $2^{1}$ | $2^{1}$ | $2^{0}$ | $2^{0}$ |
|  | $\beta$ | $\times$ | $2^{1}$ | $2^{1}$ | $2^{1}$ | $2^{1}$ |
|  | $\lambda$ | 0.001 | 0.001 | 0.001 | 0.001 | 0.001 |
| MDAv2.0-4 | $r$ | 8 | 8 | 8 | 8 | 8 |
|  | $r^{'}$ | $\times$ | $\times$ | $\times$ | 20 | 20 |
|  | $\alpha$ | $\times$ | $2^{-2}$ | $2^{-2}$ | $2^{-4}$ | $2^{-4}$ |
|  | $\beta$ | $\times$ | $2^{-2}$ | $2^{-2}$ | $2^{-2}$ | $2^{-2}$ |
|  | $\lambda$ | 0.001 | 0.001 | 0.001 | 0.001 | 0.001 |
| MDAv3.2-5 | $r$ | 3 | 3 | 3 | 3 | 3 |
|  | $r^{'}$ | $\times$ | $\times$ | $\times$ | 7 | 7 |
|  | $\alpha$ | $\times$ | $2^{-2}$ | $2^{-2}$ | $2^{-2}$ | $2^{-2}$ |
|  | $\beta$ | $\times$ | $2^{-1}$ | $2^{-1}$ | $2^{1}$ | $2^{1}$ |
|  | $\lambda$ | 0.001 | 0.001 | 0.001 | 0.001 | 0.001 |

$\times$ indicates that the benchmark model does not have this hyperparameter.

## Supplementary Parameters Analysis for TDRC


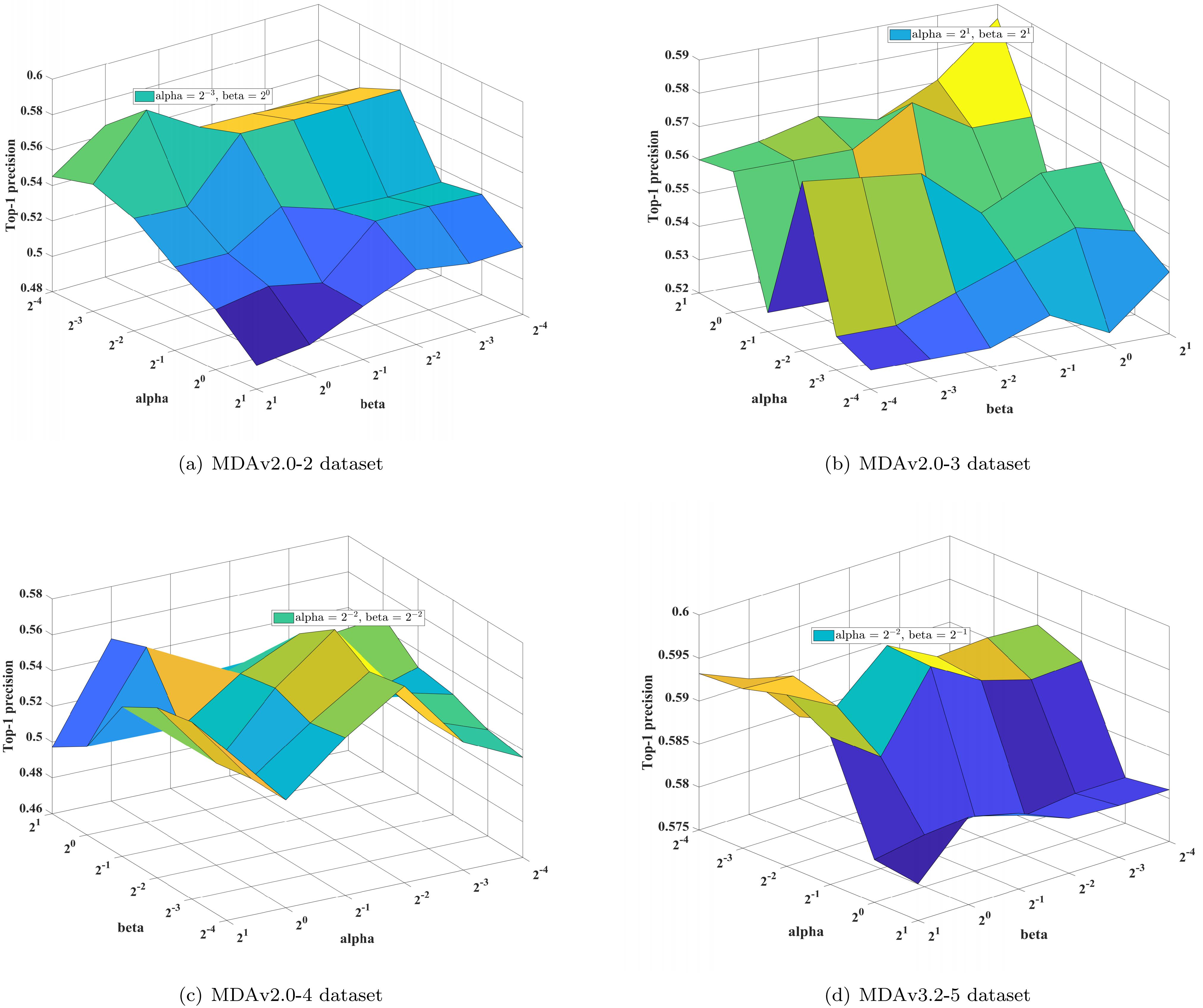


**Supplementary Figure 4.** The influence of $\alpha$ and $\beta$ on TDRC based on different datasets.

## Supplementary Parameters Analysis for TDAIGN


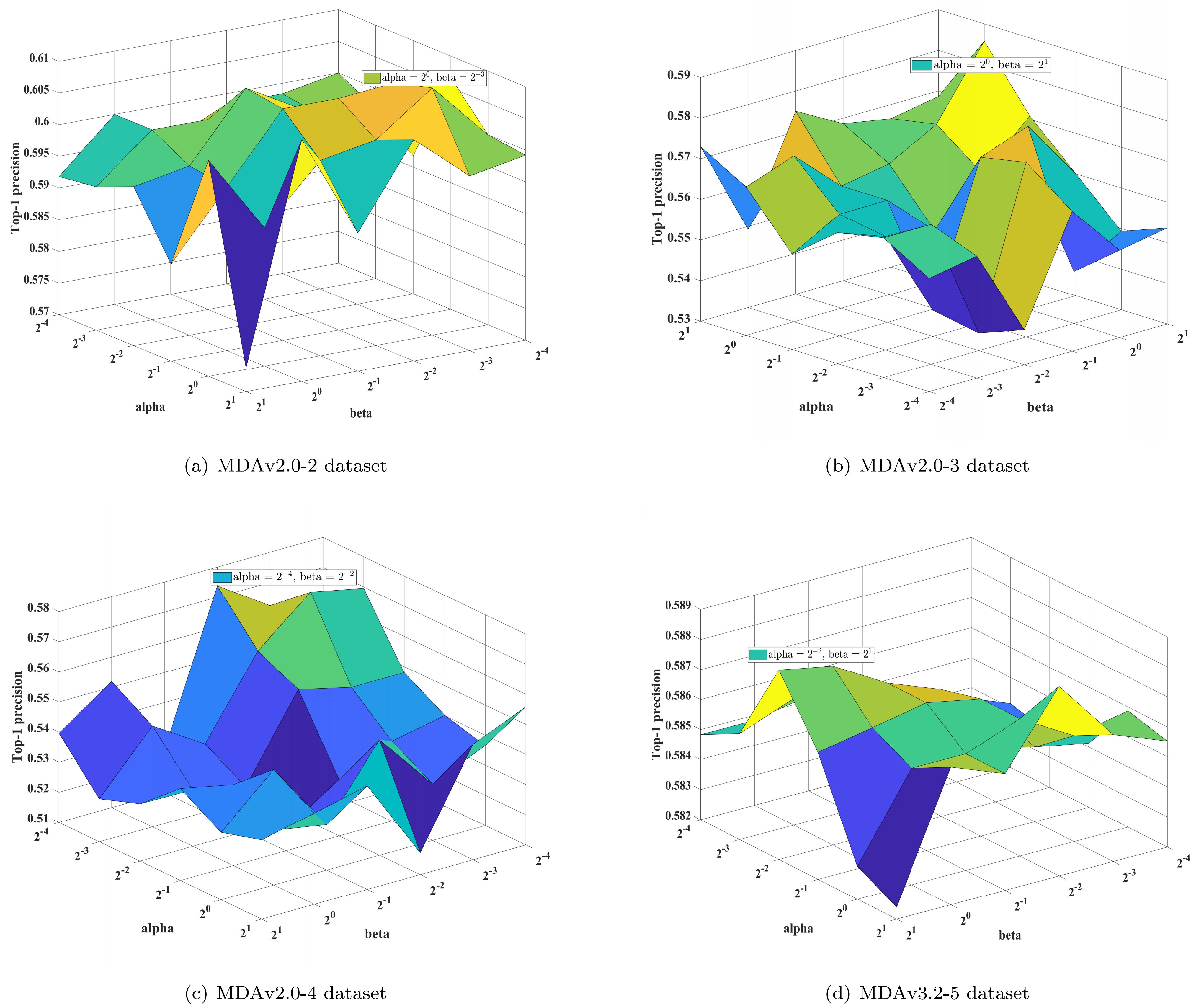


**Supplementary Figure 5.** The influence of $\alpha$ and $\beta$ on TDAIGN based on different datasets.

# References

[1] Huang, F., Yue, X., Xiong, Z., Yu, Z., Liu, S., and Zhang, W. (2021). Tensor decomposition with relational constraints for predicting multiple types of microrna-disease associations. Briefings in bioinformatics 22, bbaa140.

# Supplementary Case Studies

**Supplementary Table 2.** Top 100 disease-related miRNAs predicted by WeightTDAIGN

based on MDAv2.0-2.

|  | miRNA | disease | type | score | PMID |
| --- | --- | --- | --- | --- | --- |
| 1 | hsa-mir-21 | Breast Neoplasms | target | 1.771318 | 20346171 |
| 2 | hsa-mir-145 | Breast Neoplasms | target | 1.546713 | 22592534 |
| 3 | hsa-mir-34b | Colorectal Neoplasms | epigenetics | 1.394951 | 18519671 |
| 4 | hsa-mir-125b-1 | Carcinoma, Hepatocellular | target | 1.391683 | 25451164 |
| 5 | hsa-mir-221 | Breast Neoplasms | target | 1.389467 | 23776679 |
| 6 | hsa-mir-146a | Breast Neoplasms | genetics | 1.350276 | 18634034 |
| 7 | hsa-mir-34a | Breast Neoplasms | target | 1.335297 | 24050776 |
| 8 | hsa-mir-124-3 | Carcinoma, Hepatocellular | target | 1.294311 | 21672940 |
| 9 | hsa-mir-34c | Colorectal Neoplasms | epigenetics | 1.286871 | 18519671 |
| 10 | hsa-mir-7-3 | Colorectal Neoplasms | target | 1.283906 | 23208495 |
| 11 | hsa-mir-7-1 | Colorectal Neoplasms | target | 1.283906 | 23208495 |
| 12 | hsa-mir-7-2 | Colorectal Neoplasms | target | 1.283906 | 23208495 |
| 13 | hsa-mir-125b-2 | Carcinoma, Hepatocellular | target | 1.278873 | 22293115 |
| 14 | hsa-mir-222 | Breast Neoplasms | target | 1.274958 | 24736554 |
| 15 | hsa-mir-124-2 | Carcinoma, Hepatocellular | target | 1.256575 | 22333974 |
| 16 | hsa-mir-18a | Breast Neoplasms | genetics | 1.227822 | 16754881 |
| 17 | hsa-mir-200b | Breast Neoplasms | genetics | 1.222390 | 16754881 |
| 18 | hsa-mir-17 | Breast Neoplasms | genetics | 1.212791 | 16754881 |
| 19 | hsa-mir-16-1 | Prostatic Neoplasms | target | 1.206204 | 18931683 |
| 20 | hsa-mir-146a | Melanoma | genetics | 1.202560 | 23222547 |
| 21 | hsa-mir-1-1 | Breast Neoplasms | genetics | 1.191167 | 16754881 |
| 22 | hsa-mir-19b-1 | Carcinoma, Hepatocellular | target | 1.190857 | 26233958 |
| 23 | hsa-mir-18a | Melanoma | genetics | 1.179630 | 16754881 |
| 24 | hsa-mir-17 | Ovarian Neoplasms | genetics | 1.177823 | 16754881 |
| 25 | hsa-mir-19b-1 | Breast Neoplasms | genetics | 1.176300 | 16754881 |
| 26 | hsa-mir-19a | Breast Neoplasms | genetics | 1.171304 | 25680407 |
| 27 | hsa-mir-125b-2 | Breast Neoplasms | target | 1.166115 | 19738052 |
| 28 | hsa-mir-18a | Ovarian Neoplasms | genetics | 1.164743 | 16754881 |
| 29 | hsa-mir-17 | Melanoma | genetics | 1.154701 | 25594054 |
| 30 | hsa-mir-218-1 | Breast Neoplasms | genetics | 1.152960 | 22898079 |
| 31 | hsa-mir-133a-2 | Breast Neoplasms | genetics | 1.152117 | 16754881 |
| 32 | hsa-mir-19a | Melanoma | genetics | 1.144313 | 16754881 |
| 33 | hsa-mir-200b | Prostatic Neoplasms | target | 1.124452 | 21224847 |
| 34 | hsa-mir-19b-1 | Melanoma | genetics | 1.121150 | 16754881 |
| 35 | hsa-mir-125b-1 | Breast Neoplasms | target | 1.120255 | 20460378 |
| 36 | hsa-mir-19a | Carcinoma, Hepatocellular | target | 1.118540 | 29393488 |
| 37 | hsa-mir-218-1 | Melanoma | genetics | 1.108720 | 16754881 |
| 38 | hsa-mir-34a | Colorectal Neoplasms | target | 1.106794 | 24370784 |
| 39 | hsa-mir-200a | Breast Neoplasms | genetics | 1.106467 | 16754881 |
| 40 | hsa-mir-182 | Breast Neoplasms | target | 1.105555 | 23333633 |
| 41 | hsa-let-7a-1 | Carcinoma, Hepatocellular | target | 1.098899 | 20347499 |
| 42 | hsa-let-7a-3 | Carcinoma, Hepatocellular | target | 1.098899 | 20347499 |
| 43 | hsa-let-7a-2 | Carcinoma, Hepatocellular | target | 1.098899 | 20347499 |
| 44 | hsa-mir-218-2 | Breast Neoplasms | target | 1.093708 | Unconfirmed |
| 45 | hsa-mir-199a-2 | Breast Neoplasms | genetics | 1.092297 | 16754881 |
| 46 | hsa-mir-19a | Ovarian Neoplasms | genetics | 1.090960 | 16754881 |
| 47 | hsa-mir-488 | Breast Neoplasms | genetics | 1.082620 | 16754881 |
| 48 | hsa-mir-153-2 | Breast Neoplasms | genetics | 1.082620 | 16754881 |
| 49 | hsa-mir-215 | Breast Neoplasms | genetics | 1.081013 | 16754881 |
| 50 | hsa-mir-124-1 | Carcinoma, Hepatocellular | target | 1.073928 | 21672940 |
| 51 | hsa-let-7d | Carcinoma, Hepatocellular | target | 1.072256 | 21903590 |
| 52 | hsa-mir-25 | Breast Neoplasms | genetics | 1.072063 | 16754881 |
| 53 | hsa-mir-19b-1 | Ovarian Neoplasms | genetics | 1.070534 | 16754881 |
| 54 | hsa-mir-106b | Breast Neoplasms | genetics | 1.066023 | 16754881 |
| 55 | hsa-mir-338 | Breast Neoplasms | genetics | 1.058772 | 16754881 |
| 56 | hsa-mir-429 | Breast Neoplasms | genetics | 1.054823 | 16754881 |
| 57 | hsa-mir-18a | Carcinoma, Hepatocellular | target | 1.054184 | 19203451 |
| 58 | hsa-mir-218-2 | Stomach Neoplasms | target | 1.051502 | 20300657 |
| 59 | hsa-mir-218-1 | Stomach Neoplasms | target | 1.047267 | 20300657 |
| 60 | hsa-mir-137 | Breast Neoplasms | target | 1.045367 | 28407692 |
| 61 | hsa-mir-30c-1 | Carcinoma, Hepatocellular | target | 1.044816 | 22320217 |
| 62 | hsa-mir-30c-2 | Carcinoma, Hepatocellular | target | 1.044816 | 22320217 |
| 63 | hsa-mir-15a | Prostatic Neoplasms | target | 1.043129 | 25761682 |
| 64 | hsa-mir-16-1 | Breast Neoplasms | target | 1.042269 | 19250063 |
| 65 | hsa-mir-146a | Ovarian Neoplasms | genetics | 1.039607 | 18660546 |
| 66 | hsa-mir-218-1 | Ovarian Neoplasms | genetics | 1.033151 | 16754881 |
| 67 | hsa-mir-20a | Breast Neoplasms | target | 1.022323 | 21765466 |
| 68 | hsa-mir-367 | Breast Neoplasms | genetics | 1.020682 | 21810988 |
| 69 | hsa-mir-194-1 | Breast Neoplasms | genetics | 1.020536 | 16754881 |
| 70 | hsa-let-7b | Carcinoma, Hepatocellular | target | 1.018397 | 28671046 |
| 71 | hsa-mir-101-1 | Carcinoma, Hepatocellular | target | 1.015438 | 26718325 |
| 72 | hsa-mir-20a | Colorectal Neoplasms | target | 1.012381 | 21242194 |
| 73 | hsa-let-7g | Carcinoma, Hepatocellular | target | 1.012368 | 20309945 |
| 74 | hsa-mir-16-1 | Carcinoma, Hepatocellular | target | 1.009801 | 23226427 |
| 75 | hsa-mir-383 | Breast Neoplasms | genetics | 1.007226 | 16754881 |
| 76 | hsa-mir-214 | Breast Neoplasms | genetics | 1.005061 | 27328731 |
| 77 | hsa-mir-128-1 | Breast Neoplasms | target | 1.004523 | 21953503 |
| 78 | hsa-mir-106b | Melanoma | genetics | 1.004520 | 16754881 |
| 79 | hsa-mir-128-2 | Breast Neoplasms | target | 1.004226 | 23526655 |
| 80 | hsa-mir-25 | Melanoma | genetics | 1.002829 | 16754881 |
| 81 | hsa-mir-195 | Breast Neoplasms | target | 1.002503 | 16754881 |
| 82 | hsa-mir-135b | Breast Neoplasms | genetics | 1.001408 | 16754881 |
| 83 | hsa-let-7e | Carcinoma, Hepatocellular | target | 0.999612 | 20347499 |
| 84 | hsa-mir-93 | Breast Neoplasms | genetics | 0.998525 | 16754881 |
| 85 | hsa-mir-148a | Colorectal Neoplasms | target | 0.997121 | 21455217 |
| 86 | hsa-let-7f-2 | Carcinoma, Hepatocellular | target | 0.990149 | 20347499 |
| 87 | hsa-let-7f-1 | Carcinoma, Hepatocellular | target | 0.990149 | 21903590 |
| 88 | hsa-mir-302a | Breast Neoplasms | genetics | 0.990147 | 16754881 |
| 89 | hsa-mir-339 | Breast Neoplasms | genetics | 0.987145 | 16754881 |
| 90 | hsa-mir-302d | Breast Neoplasms | genetics | 0.986457 | 16754881 |
| 91 | hsa-mir-20a | Ovarian Neoplasms | genetics | 0.983491 | 22235027 |
| 92 | hsa-mir-30d | Breast Neoplasms | genetics | 0.980613 | 16754881 |
| 93 | hsa-mir-302b | Breast Neoplasms | genetics | 0.980399 | 16754881 |
| 94 | hsa-mir-302c | Breast Neoplasms | genetics | 0.980398 | 16754881 |
| 95 | hsa-mir-214 | Carcinoma, Hepatocellular | target | 0.979843 | 22359598 |
| 96 | hsa-mir-124-1 | Colorectal Neoplasms | epigenetics | 0.979729 | 21327300 |
| 97 | hsa-mir-320a | Breast Neoplasms | genetics | 0.979273 | 16754881 |
| 98 | hsa-mir-30b | Breast Neoplasms | genetics | 0.978148 | 16754881 |
| 99 | hsa-mir-143 | Colorectal Neoplasms | target | 0.976728 | 28619512 |
| 100 | hsa-mir-124-3 | Colorectal Neoplasms | epigenetics | 0.976640 | 21327300 |

**Supplementary Table 3.** Top 100 disease-related miRNAs predicted by WeightTDAIGN

based on MDAv2.0-3.

|  | miRNA | disease | type | score | PMID |
| --- | --- | --- | --- | --- | --- |
| 1 | hsa-mir-210 | Breast Neoplasms | circulation | 1.311595 | 22370716 |
| 2 | hsa-mir-221 | Breast Neoplasms | target | 1.278975 | 18708351 |
| 3 | hsa-mir-200c | Carcinoma, Hepatocellular | epigenetics | 1.26926 | 23222811 |
| 4 | hsa-mir-17 | Ovarian Neoplasms | genetics | 1.265794 | 16754881 |
| 5 | hsa-mir-17 | Melanoma | genetics | 1.259342 | 16754881 |
| 6 | hsa-mir-34b | Colorectal Neoplasms | epigenetics | 1.253215 | 18519671 |
| 7 | hsa-mir-200a | Prostatic Neoplasms | target | 1.241373 | 21224847 |
| 8 | hsa-mir-124-1 | Colorectal Neoplasms | epigenetics | 1.239199 | 21327300 |
| 9 | hsa-mir-200b | Breast Neoplasms | genetics | 1.236993 | 16754881 |
| 10 | hsa-mir-7-3 | Colorectal Neoplasms | target | 1.226711 | 23208495 |
| 11 | hsa-mir-7-1 | Colorectal Neoplasms | target | 1.226711 | 23208495 |
| 12 | hsa-mir-7-2 | Colorectal Neoplasms | target | 1.226711 | 23208495 |
| 13 | hsa-mir-34c | Colorectal Neoplasms | epigenetics | 1.218591 | 18519671 |
| 14 | hsa-mir-196a-2 | Carcinoma, Hepatocellular | epigenetics | 1.21581 | 21692953 |
| 15 | hsa-mir-145 | Breast Neoplasms | target | 1.192128 | 19360360 |
| 16 | hsa-mir-218-2 | Stomach Neoplasms | target | 1.187551 | 20300657 |
| 17 | hsa-mir-126 | Breast Neoplasms | target | 1.179025 | 21249429 |
| 18 | hsa-mir-200c | Stomach Neoplasms | target | 1.176263 | 25986864 |
| 19 | hsa-mir-200b | Carcinoma, Hepatocellular | epigenetics | 1.173081 | 22370893 |
| 20 | hsa-mir-34a | Urinary Bladder Neoplasms | target | 1.161175 | 22684561 |
| 21 | hsa-mir-218-1 | Melanoma | genetics | 1.160623 | 16754881 |
| 22 | hsa-mir-218-1 | Stomach Neoplasms | target | 1.153434 | 20300657 |
| 23 | hsa-mir-125b-1 | Carcinoma, Hepatocellular | target | 1.145017 | 22293115 |
| 24 | hsa-mir-124-3 | Carcinoma, Hepatocellular | target | 1.144505 | 21672940 |
| 25 | hsa-mir-221 | Stomach Neoplasms | target | 1.140762 | 19153141 |
| 26 | hsa-mir-101-1 | Carcinoma, Hepatocellular | target | 1.123656 | 26718325 |
| 27 | hsa-mir-34a | Breast Neoplasms | target | 1.122359 | 21814748 |
| 28 | hsa-mir-124-1 | Breast Neoplasms | target | 1.121571 | 22085528 |
| 29 | hsa-mir-1-1 | Carcinoma, Hepatocellular | target | 1.119292 | 22664953 |
| 30 | hsa-mir-181a-2 | Breast Neoplasms | circulation | 1.118574 | 22692639 |
| 31 | hsa-mir-124-2 | Colorectal Neoplasms | epigenetics | 1.118474 | 21327300 |
| 32 | hsa-mir-200b | Prostatic Neoplasms | target | 1.115172 | 21224847 |
| 33 | hsa-mir-141 | Breast Neoplasms | target | 1.112813 | 25813250 |
| 34 | hsa-mir-18a | Carcinoma, Hepatocellular | target | 1.111593 | 28399983 |
| 35 | hsa-mir-124-2 | Carcinoma, Hepatocellular | target | 1.109143 | 21672940 |
| 36 | hsa-mir-17 | Breast Neoplasms | genetics | 1.106038 | 19048628 |
| 37 | hsa-mir-218-1 | Ovarian Neoplasms | genetics | 1.105633 | 16754881 |
| 38 | hsa-mir-124-3 | Colorectal Neoplasms | epigenetics | 1.09891 | 21327300 |
| 39 | hsa-mir-199a-2 | Breast Neoplasms | genetics | 1.090193 | 16754881 |
| 40 | hsa-mir-34b | Urinary Bladder Neoplasms | epigenetics | 1.085195 | 21225432 |
| 41 | hsa-mir-27a | Breast Neoplasms | target | 1.079238 | 24517586 |
| 42 | hsa-mir-124-2 | Breast Neoplasms | target | 1.078074 | 22085528 |
| 43 | hsa-mir-200c | Breast Neoplasms | epigenetics | 1.075807 | 23112837 |
| 44 | hsa-mir-18a | Melanoma | genetics | 1.07577 | 16754881 |
| 45 | hsa-mir-34b | Breast Neoplasms | epigenetics | 1.074193 | 21225432 |
| 46 | hsa-mir-195 | Breast Neoplasms | target | 1.072794 | 22328513 |
| 47 | hsa-mir-133a-2 | Breast Neoplasms | genetics | 1.071021 | 22292984 |
| 48 | hsa-mir-19b-1 | Melanoma | genetics | 1.064682 | 16754881 |
| 49 | hsa-mir-9-1 | Breast Neoplasms | genetics | 1.062764 | 16754881 |
| 50 | hsa-mir-34a | Colorectal Neoplasms | target | 1.060512 | 24370784 |
| 51 | hsa-mir-145 | Urinary Bladder Neoplasms | target | 1.05755 | 22108519 |
| 52 | hsa-mir-181a-1 | Breast Neoplasms | circulation | 1.05674 | Unconfirmed |
| 53 | hsa-mir-145 | Carcinoma, Hepatocellular | target | 1.055321 | 23499894 |
| 54 | hsa-mir-125b-2 | Breast Neoplasms | target | 1.054142 | 22307404 |
| 55 | hsa-mir-15a | Prostatic Neoplasms | target | 1.052757 | 25761682 |
| 56 | hsa-mir-29b-2 | Breast Neoplasms | target | 1.051074 | 22330642 |
| 57 | hsa-mir-19b-1 | Ovarian Neoplasms | genetics | 1.050742 | 22235027 |
| 58 | hsa-mir-31 | Breast Neoplasms | target | 1.045143 | 23364795 |
| 59 | hsa-mir-200a | Breast Neoplasms | genetics | 1.043136 | 16754881 |
| 60 | hsa-mir-29b-1 | Breast Neoplasms | target | 1.041333 | 22330642 |
| 61 | hsa-mir-19a | Melanoma | genetics | 1.039838 | 16754881 |
| 62 | hsa-mir-18a | Ovarian Neoplasms | genetics | 1.035054 | 16754881 |
| 63 | hsa-mir-125b-2 | Carcinoma, Hepatocellular | target | 1.031995 | 22293115 |
| 64 | hsa-mir-17 | Carcinoma, Hepatocellular | target | 1.031878 | 23418359 |
| 65 | hsa-mir-20a | Breast Neoplasms | target | 1.030109 | 26829385 |
| 66 | hsa-mir-125b-1 | Breast Neoplasms | target | 1.02401 | 25451164 |
| 67 | hsa-mir-29b-1 | Carcinoma, Hepatocellular | target | 1.014844 | 21625215 |
| 68 | hsa-mir-16-1 | Prostatic Neoplasms | target | 1.013256 | 18931683 |
| 69 | hsa-mir-1-1 | Prostatic Neoplasms | target | 1.013033 | 22068816 |
| 70 | hsa-mir-1-1 | Breast Neoplasms | genetics | 1.011292 | 16754881 |
| 71 | hsa-mir-218-1 | Breast Neoplasms | target | 1.009396 | Unconfirmed |
| 72 | hsa-mir-199a-1 | Urinary Bladder Neoplasms | target | 1.003923 | 21807947 |
| 73 | hsa-mir-92a-1 | Breast Neoplasms | circulation | 0.999589 | 23052693 |
| 74 | hsa-mir-19a | Ovarian Neoplasms | genetics | 0.996518 | 16754881 |
| 75 | hsa-mir-200a | Carcinoma, Hepatocellular | epigenetics | 0.995934 | 21837748 |
| 76 | hsa-mir-124-3 | Breast Neoplasms | genetics | 0.994579 | 21318219 |
| 77 | hsa-mir-18a | Breast Neoplasms | genetics | 0.993036 | 16754881 |
| 78 | hsa-mir-148a | Colorectal Neoplasms | epigenetics | 0.97198 | 21327300 |
| 79 | hsa-mir-200c | Prostatic Neoplasms | target | 0.967879 | 22370643 |
| 80 | hsa-mir-34b | Stomach Neoplasms | epigenetics | 0.967595 | 21213213 |
| 81 | hsa-mir-7-2 | Urinary Bladder Neoplasms | target | 0.965278 | 23742934 |
| 82 | hsa-mir-7-3 | Urinary Bladder Neoplasms | target | 0.965278 | 23742934 |
| 83 | hsa-mir-7-1 | Urinary Bladder Neoplasms | target | 0.965278 | 23742934 |
| 84 | hsa-mir-199a-2 | Colorectal Neoplasms | target | 0.959747 | 22674437 |
| 85 | hsa-mir-34b | Pancreatic Neoplasms | epigenetics | 0.958471 | 21225432 |
| 86 | hsa-mir-101-2 | Carcinoma, Hepatocellular | target | 0.955143 | 19133651 |
| 87 | hsa-mir-218-2 | Breast Neoplasms | target | 0.951005 | Unconfirmed |
| 88 | hsa-mir-19a | Breast Neoplasms | genetics | 0.947792 | 16754881 |
| 89 | hsa-mir-124-1 | Carcinoma, Hepatocellular | target | 0.946091 | 21672940 |
| 90 | hsa-mir-199a-1 | Carcinoma, Hepatocellular | target | 0.94208 | 21807947 |
| 91 | hsa-mir-196a-2 | Breast Neoplasms | genetics | 0.939146 | 18634034 |
| 92 | hsa-mir-101-2 | Breast Neoplasms | target | 0.938992 | 23071542 |
| 93 | hsa-mir-29c | Breast Neoplasms | target | 0.936275 | 24577056 |
| 94 | hsa-mir-19b-1 | Breast Neoplasms | genetics | 0.934765 | 25680407 |
| 95 | hsa-mir-328 | Breast Neoplasms | target | 0.93085 | 19270061 |
| 96 | hsa-mir-34a | Prostatic Neoplasms | target | 0.927688 | 21240262 |
| 97 | hsa-mir-199a-2 | Urinary Bladder Neoplasms | target | 0.922212 | 21807947 |
| 98 | hsa-mir-1-1 | Urinary Bladder Neoplasms | target | 0.92032 | 21304530 |
| 99 | hsa-mir-34b | Carcinoma, Non-Small-Cell Lung | epigenetics | 0.917025 | 21702040 |
| 100 | hsa-mir-205 | Breast Neoplasms | target | 0.9168 | 19276373 |
